# Human gene and disease associations for clinical‐genomics and precision medicine research

**DOI:** 10.1002/ctm2.28

**Published:** 2020-05-03

**Authors:** Zeeshan Ahmed, Saman Zeeshan, Dinesh Mendhe, XinQi Dong

**Affiliations:** ^1^ Institute for Health, Health Care Policy and Aging Research, Rutgers The State University of New Jersey New Brunswick New Jersey USA; ^2^ Department of Medicine, Rutgers Robert Wood Johnson Medical School Rutgers Biomedical and Health Sciences New Brunswick New Jersey USA; ^3^ Rutgers Cancer Institute of New Jersey, Rutgers The State University of New Jersey New Brunswick New Jersey USA

**Keywords:** clinical‐genomics, database, diseases, drugs, genes, germline mutations, precision medicine, somatic mutations

## Abstract

We are entering the era of personalized medicine in which an individual's genetic makeup will eventually determine how a doctor can tailor his or her therapy. Therefore, it is becoming critical to understand the genetic basis of common diseases, for example, which genes predispose and rare genetic variants contribute to diseases, and so on. Our study focuses on helping researchers, medical practitioners, and pharmacists in having a broad view of genetic variants that may be implicated in the likelihood of developing certain diseases. Our focus here is to create a comprehensive database with mobile access to all available, authentic and actionable genes, SNPs, and classified diseases and drugs collected from different clinical and genomics databases worldwide, including Ensembl, GenCode, ClinVar, GeneCards, DISEASES, HGMD, OMIM, GTR, CNVD, Novoseek, Swiss‐Prot, LncRNADisease, Orphanet, GWAS Catalog, SwissVar, COSMIC, WHO, and FDA. We present a new cutting‐edge gene‐SNP‐disease‐drug mobile database with a smart phone application, integrating information about classified diseases and related genes, germline and somatic mutations, and drugs. Its database includes over 59 000 protein‐coding and noncoding genes; over 67 000 germline SNPs and over a million somatic mutations reported for over 19 000 protein‐coding genes located in over 1000 regions, published with over 3000 articles in over 415 journals available at the PUBMED; over 80 000 ICDs; over 123 000 NDCs; and over 100 000 classified gene‐SNP‐disease associations. We present an application that can provide new insights into the information about genetic basis of human complex diseases and contribute to assimilating genomic with phenotypic data for the availability of gene‐based designer drugs, precise targeting of molecular fingerprints for tumor, appropriate drug therapy, predicting individual susceptibility to disease, diagnosis, and treatment of rare illnesses are all a few of the many transformations expected in the decade to come.

## 1 INTRODUCTION

Since the beginning of scientific discoveries, it has been critically central to understand the cause of disease, pain, and senescence.^1^ Over the centuries, quests for the answers have led us to take giant leaps. It was only in the last century that the discovery of antibiotics freed us from many of the dreaded diseases of the past. Today, we stand on the threshold of the new medical revolution, just as big and far‐reaching. Despite of all our scientific knowledge, much of medicine today is still based on the conventional symptomatic treatment and performing learned trials for symptom relief, which works for most patients but not all. The current medical model is based on disease classification, which is routinely composed of data from healthcare units brought from different streams, which includes imaging, pathology, genomics, electrophysiology, and others.^2^ Treating on symptomatic basis can be complex for multifaceted and multisymptomatic conditions. However, genetic research can assist in producing individual treatment solutions, rather than what works for the average person, and with a precise understanding of who is at risk for critical diseases like diabetes, high blood pressure, or cancer. This will bring systematic approach to healing, allowing for rapid disease detection at an early stage, accurate characterization of disease, and assign preventive measures needed before the disease even appears. Also, timely discovery and association of genetic variants with diseases will help develop a more effective therapy tailored to an individual's precise genetic makeup, reducing adverse drug reactions. As biological data accumulates at larger scales and at exponential rates, because of higher‐throughput and lower‐cost DNA sequencing technologies, it has become essential to develop innovative, smart, and modern bioinformatics applications to help improve research quality and data sharing. New tools will provide a progressive understanding of heterogeneous genomics and clinical findings and facilitate increases in clinical utilization of information in these databases and translation to healthcare.

It has been over 100 years since the term “Gene” was introduced,^3^ and its meaning has progressively evolved in several scientific directions.^1^, ^4^, ^5^ A gene is a unit of hereditary, made up of segment of DNA sequence that carries genetic information, which defines a biological function.^6^ The chemical structure of the genome is double‐stranded DNA, and the smallest unit of genetic information is the base pair (bp), which is two nucleotides paired by hydrogen bonds across the double helix. The human genome contains 3 billion bps that form tens of thousands of genes, yet its complex structure is made up of only four molecules: adenine (A), cytosine (C), guanine (G), and thymine (T).^7^ Most genes direct our cells to make a specific protein necessary for some function, and collections of proteins with other organic molecules perform all the tasks needed for life, for example, cell signaling, building body structures, and fighting diseases. Most human genes have a discontinuous structure, with the protein coding regions, or exons, interrupted by noncoding regions, or introns.^8^ An average human gene has nine exons, and the longest known human gene called titin (TTN) has 365 exons spanning 109 224 bp and encodes a protein comprising 35 991 amino acids.^9^ For a long period of time, researchers used a broad estimate of gene count at more than 50 000 genes, including 21 000 protein‐coding genes.^10^ However, this number has been repeatedly overturned with advancements in genetics and genomics research.

The completion of the Human Genome Project^11^, ^12^ was a major scientific development in human genomics and biomedical sciences. Its findings suggested that all humans are 99.9% genetically identical and only 0.1% of genetic variations are responsible for the phenotypic differences, such as physical traits (eg, height, intelligence, hair, and eye color), disease susceptibility, and drug responses, among individuals in populations.^13^ The human genome harbors enormous gene‐encoded protein diversity and expression differences, and variability between individuals due to DNA polymorphisms that underlay the differences between us, which importantly can impart susceptibility to certain diseases and resistance to medications. A major goal of medical genetics is to identify genes that when altered lead to human disease, but not all‐recognizable DNA sequence alterations result in disease.^14^ Most alterations, or mutations, are simple differences, such as restriction fragment length polymorphisms, single nucleotide polymorphisms (SNPs), that may not change the expression or coding of a gene, but some specific mutations can change gene instructions, and ultimately create a protein malfunction, which may cause disease. Human variability is dynamic and fundamental to the survival and advancement of humans. Genetic variations are the differences in DNA sequence within the genome of individuals in populations.^15^ Their discovery dates back to 1950s when association studies to link specific variants in biological candidate genes to disease risk emerged.^16^ If we can identify which genetic variations are associated with specific diseases, we will be better equipped to find new treatments and even cures. Also identifying genetic variations will help us better understand why some people respond differently to similar medications. Comparisons of frequencies of genetic variants among affected and unaffected individuals revealed correlations between blood‐group antigens and peptic ulcer disease^17^; in the 1960s and 1970s, common variation at the human leukocyte antigen (HLA) locus was associated with autoimmune and infectious diseases^18^; and in the 1980s, apolipoprotein E was implicated in the etiology of Alzheimer's disease.^19^ Still, only about a dozen extensively reproduced associations of common variants were identified in the 20th century.^20^

In general, mutations can be grouped into two different types: germline and somatic.^20^ Germline mutations are variations found in all cells of an organism, including germ line cells. They play an important role in evolution by giving every human its unique genetic makeup but also give rise to hereditary diseases. Somatic mutations are not inherited but acquired during lifetime in somatic cells of an organism and might cause tissue‐specific tumors. They are present in the genomes of all dividing cells, both normal and neoplastic. They occur as a result of misincorporation during DNA replication or through exposure to exogenous or endogenous mutagens.^21^ Cancer, as a disease of genome alterations, arises through the sporadic acquisition of multiple somatic mutations.^22^ All cancers arise as a result of the acquisition of these abnormalities, including base substitutions, deletions, amplifications, and rearrangements. The extent to which each of these mechanisms contributes to cancer varies markedly between different genes, and also between different cancer types.

Early efforts to analyze common variations in the genome were hampered by lack of a reference genome and cost‐effective means of identifying, or genotyping, SNPs.^23^ In the early 2000s, cheaper genotyping technologies enabled the availability of vast number of SNPs, which provided the first needed map with information about the genetic relatedness of SNPs by The SNP Consortium^24^ and launched the HapMap project of human genetic variation.^25^ Today, genetic mutations responsible for thousands of conditions, such as cancer, hypertension, and heart diseases, have been identified by scientists. These associations cannot be easily deciphered, because they are often impacted by interactions between dozens of different genes, many of which are caused by single gene elements and the environment. To identify these complex and widely prevalent elements, scientists may have to profile the genetic signatures of thousands of people, even multiple populations, and not just a few individuals. This high density of genotype data allows for unraveling the clinical and therapeutic relevance of genetic variants. The genomic and epigenomic (chemically modified genome)^26^ interpretation has led to the fundamentals of development and progression of human diseases,^27^ categorized as multifactorial, mitochondrial,^28^ chromosomal,^29^ and monogenic^30^ diseases. All human disease classifications are maintained by the World Health Organization (WHO) with the standard creation of International Classification of Disease (ICD) codes and their related medications organized by the Food and Drug Administration (FDA) in the form of National Drug Code (NDC) codes (applicable in the United States). With the emergence of new‐generation gene sequencing techniques, numerous databases have surfaced for gene annotation. They are accessible through web and desktop interfaces and claim to provide information about genes and related diseases, for example, Disease Ontology,^31^ DiseaseEnhancer,^32^ DISEASES,^33^ DisGeNET,^34^ eDGAR,^35^ GeneCard,^36^ Genetic Testing Registry (GTR),^37^ MalaCard,^38^ Online Mendelian Inheritance in Man (OMIM),^39^ miR2Disease,^40^ Human Gene Mutation Database (HGMD),^41^ Disease Network Database (DNetDB),^42^ ClinVar,^43^ Orphanet,^44^ Gene2Function,^45^ Swiss‐Prot,^46^ LncRNADisease,^47^ Lnc2Cancer v.20,^48^ and so on. These databases are useful, but none of them cover all the currently available genome, classified diseases and drugs data in a standardized, integrated, and annotated format.^1^

Time‐to‐time technological advancements have heavily revolutionized the field of genomics, especially when it is about, for example, triple code development, gene number proposition, genetic mapping, data banks, gene‐disease maps, catalogues of human genes and genetic disorders, big data, and next‐generation sequencing (NGS).^49^ With advancements at such an exponential pace, innovative, smart, and modern bioinformatics applications are necessary to help improve the quality and transition of healthcare. Artificial intelligence (AI) and internet of things (IoT) is becoming one of the landmark developments in personalized and public health.^50^, ^51^ Millions of AI‐ and IoT‐based medical devices (eg, sensors, actuators, smartphones, tablets, wearable devices, laptops, etc) are in use worldwide, with effective responses.^52^ Its applications include patient's activity tracking systems,^53^, ^54^ monitoring physiological signals (eg, heart rate, electrocardiography, body temperature, position, and blood pressure),^55^ observing environmental conditions^56^ and object detection,^57^, ^58^ and many more. Still, there is no AI‐ and IoT‐based solution available, which can facilitate precision medicine and clinical genomics research with detailed information about all available and actionable genes, somatic and germline mutations, and integrating ICD and NDC lists in an effective way. Millions of people worldwide getting their DNA sequenced and would like to have information about their genomics profiles, which includes information about their genes and mutations, when most of the people are unable to interpret such information. We believe that our study and developed application is a potential solution to fill this information gap by helping people with the provision of high‐quality information associated with their genome and clinical profiles.

The convergence of science, medicine, and information technology today has created a unique possibility to manage our human health in new ways. As Apple's relevance in the healthcare space grows, the iPhone is becoming an integral part in advancing the fundamental science by unfolding the complexities of disease biology and understanding of the human genome variations, making them accessible through smart devices. Carrying this goal forward, we present to the biomedical research community, a mobile database for iOS smartphone and tablet devices for simple, easy‐to‐use access to the information based on all available genes and SNPs collected from the sequenced human genome, and their relevant diseases and drugs data. It is another milestone towards achieving the goals of personalized medicine, as it will bring swift excess to gene and mutation data in genomic research providing scientists and clinicians with variant‐by‐variant information of mutations that altered coding instructions of genes. App's interactive infographics let researchers see at a glance all mutations reported for the input gene, and its corresponding gene, mutation, disease, drug and study information, and its efficient querying ability offers the user with an important knowledge discovery tool, just a click away.

Extensive growth of biological data with an increasing number of developed biological databases aims to assist human research in drawing pathways leading from genes to disease depending on the variable data types. Most of the available databases are not timely updated and do not intuitively give corresponding disease information but require users to constantly shuttle between different pages and options until they find the most appropriate results.^59^, ^60^ Not all databases and tools specifically focus on the relationship between genes, SNPs, diseases, and drugs.^61^, ^62^, ^63^ One platform that has proven to be an efficient tool in several areas, including healthcare, is the smartphone application.^47^, ^48^ As smart devices have become increasingly popular, there is still no iOS app publicly available that can provide unified access to genomic and healthcare databases with easy navigation and free portable access to all available genes, germline and somatic mutations, and related diseases and drugs for efficient and robust classifications. Our focus is to create a comprehensive database with mobile access to authentic and actionable genes, SNPs, and classified diseases and drugs, considering the foundation for clinical and genomics research, pathology, epidemiology, and precision medicine. Overall study is divided into following three aims: healthcare database development of classified diseases and drugs with their medical codes; genomics database implementation of authentic and actionable genes and variants, and their annotation with diseases; and iOS app development for mobile access to individualized and annotated information. Our research aims include centralized and annotated gene‐SNP‐disease‐drug database, which will not only store, organize, and share data in a structured and searchable manner but will also facilitate data retrieval with an iOS application for iPhone and iPad. The greatest strength of our approach is unearthing the biological roots of complex and rare diseases. It is a comprehensive approach to facilitate searching for unknown disease variants that have not previously been associated with their respective diseases. To harness the power of genes, SNPs, and other variants reported, our presented solution will contribute as a state‐of‐the‐art leading mobile application, for the research community for gene‐variant search. It is a powerful application to address the needs of clinical research to uncover previously unsuspected, yet important, biological variants, mechanisms, and pathways that could be potentially targeted with drugs.

## 2 METHODS AND MATERIALS

NGS advancements have facilitated and accelerated the process of identifying genetic variations. Adoption of NGS with whole‐genome and RNA sequencing in a diagnostic context has the potential to improve disease risk detection in support of precision medicine and drug discovery. Several bioinformatics pipelines have been developed to strengthen variant interpretation by efficiently processing and analyzing sequenced data, whereas many published results show how genomic data can be proactively incorporated into medical practices and raise utilization of clinical information. Innovative and smart systems are necessary to help improve the quality and transition of healthcare by studying heterogeneous healthcare and genomics data together.

Our focus here was to develop a well‐structured, comprehensive, centralized, and integrated gene‐SNP‐disease‐drug database, which stores, organizes, and shares data in a structured and searchable manner but facilitates data retrieval with smartphone application and provides visualization features for analytics. Database includes all available and actionable genes collected from global sources, including those approved by The American College of Medical Genetics and Genomics (ACMG)^64^ and Memorial Sloan Kettering (MSK) IMPACT,^65^ germline and somatic mutations maintained by the Genome‐wide Association Studies (GWAS)^23^ Catalog (The NHGRI‐EBI Catalog of published genome‐wide association studies) and Catalogue Of Somatic Mutations In Cancer (COSMIC),^66^, ^67^ classified diseases by the WHO, and drugs maintained by the FDA. In this study, we present PAS, a nonprofit, academic, and publicly available iOS app, which invites global users to freely download it on iPhone & iPad devices, quickly adopt its easy to use interface, and search for genes, SNPs, and related diseases and drugs. Presented app provides smart distillation (data cleansing and restructuring) and abundant distribution (involvement of a gene in multiple disease pathways and phenotypes) of genes associated with classified diseases, supporting both scientists and providers with greater emphasis and easy one‐tap browsing, saving time in scanning through genes, SNPs, and developing gene‐SNP‐disease lists for a research study. It promotes interoperability (multiplatform usage) and comparability (multiple ICD codes for a disease phenotype) in data presentation of genes, SNPs, and ICD codes to map health conditions to their corresponding diseases, to benefit every type of user (eg, researchers, medical practitioners, pharmacists, life science students, or even patients).

On a macro level, the more accurate data are, the better is the clinical assessment and patient management for the greater good of public health, allowing to track and respond to global health threats faster and compare best practices with the international community. Database of the app involves medical classified codes, disease, drugs, and related information, such as signs and symptoms, abnormal findings, complaints, social circumstances, and external causes of injury. Such information is supplementary part of created relational database of PAS, which mainly includes information about WHO‐maintained 13 523 ICD‐9 and 70 663 ICD‐10, and FDA‐approved 123 701 NDC codes (Table 1). Having detailed information associated with medical codes is very helpful, especially when a user is a medical professional and relating with medical records of patient for specific conditions. Initial genomics data includes standard human reference genome and disease‐gene‐variant data collected from different genomics databases worldwide, including ClinVar, GeneCards, DISEASES, HGMD, OMIM, GTR, CNVD, Ensembl, GenCode, Novoseek (http://www.novoseek.com), Swiss‐Prot, LncRNADisease, Orphanet, LincSNP 2.0,^68^ MiRNA SNP Disease Database (MSDD),^69^ COSMIC, and GWAS Catalog

**TABLE 1:** Database description and statistics of ICD codes

Categories	Count
Total diagnosis codes	84 186
Total ICD 10 codes	70 663
Total ICD 9 codes	13 523
Distinct diseases	82 384
Distinct diseases based on ICD 10 codes	70 629
Distinct diseases based on ICD 9 codes	13 518

The criteria for selecting genomics databases included information about genes and associated diseases. We were mainly interested in finding, if any clinical genomics database is available, which provides information about all available and actionable genes and link those to classified diseases and their ICD codes maintained by the WHO. We had to adopt time‐consuming and laborious data evaluation and extraction process. We first learned about available databases, their associated online websites and tools, file formats, and data structures.^1^ We created individual lists of data extracted from all the sources, and written different data parsers to classify data and upload data in to our database. We have included not only protein coding genes but also functional RNAs as they provide great resources of related diseases.

Our compiled genes dataset (includes Gencode release 29) consists of total 59 293 genes (19 989 are protein coding and 39 304 are nonprotein coding) (Table 2). The nonprotein coding genes are of 24 different types (processed transcript, lincRNA, antisense, immunoglobulin genes, bidirectional promoter lncRNA, polymorphic pseudogene, transcribed unitary pseudogene, transcribed unprocessed pseudogene, transcribed processed pseudogene, sense overlapping, scRNA, noncoding, unprocessed pseudogene, unitary pseudogene, vaultRNA, TRC gene, sense intronic, snRNA, processed pseudogene, to be experimentally confirmed genes, T cell receptor genes, pseudogenes, and macro lncRNA) located at 23 pairs of genomic chromosomes and mitochondrial DNA, and with over 200 000 gene‐disease combinations. Germline mutation dataset includes over 67 000 SNPs reported for over 19 000 genes, located in over 1000 regions, published with over 3000 articles in over 415 journals available in the PUBMED, and over 100 000 classified SNP‐disease combinations, whereas somatic mutation dataset encompasses human genes with over 15 million SNPs coding mutations across over a million samples (Table 3). Total of 223 key cancer census genes have been subjected to deep, exhaustive curation by expert scientists, gathering information from 26 251 papers to date,^66^ merged with genome‐wide annotations from 466 whole genome and large‐scale systematic screens publications, as well as open access data from The Cancer Genome Atlas^70^ and the International Cancer Genome Consortium.^71^ All the reported figures have been calculated by querying our PAS database using SQL, where all the relevant information extracted from various sources is restructured and integrated among normalized relations.

**TABLE 2:** Gene database description and statistics

Categories	Count
Genes‐disease combinations	98 064
Gene types	26
Chromosomes	24
Genes (including aliases)	13 216
Genes (Ensembl IDs)	10 598
Unique diseases	12 257
Genes‐disease combinations based on actionable genes	32 089
Distinguished genes‐disease source combinations	809
Cancer leading genes	8063

**TABLE 3:** SNP database description and statistics

Categories	Count
SNP‐disease combinations	101 439
SNPs	67 727
Strongest SNP risk allele	73 070
SNP gene IDs	13 979
Reported genes	19 669
Regions	1045
Disease traits	3041
Literature (PUBMED articles)	3186
Contexts	119

### 2.1 Healthcare database development of classified diseases and drugs

Electronic health record (EHR) operational and analytical systems depend on two main clinical entities: diagnoses and medications.^72^, ^73^ The first medical classification system was released in 1700, and underwent a long evolution through the late 18th century until 1948, when the WHO took responsibility with the classification of the ICD codes.^72^, ^73^, ^74^ To avoid redundancies and misinterpretations, both are defined and standardized by medical professionals through universally assigned lists of clinical codes based on ICDs. The clinical code‐sets offer substantial clinical value to healthcare, which includes new diagnoses and treatments in clinical research and population health. These codes are entered in the EHR system by the medical professionals.^74^ WHO designs, maintains, and updates the ICD codes to standardize nomenclature, hierarchically systematize, categorize, and structure disease names in medicine. ICD‐9 is still active, which will eventually be replaced by the 10th or later 11th version. Both ICD‐9 and ICD‐10 versions are currently used for administrative and public health statistics, medical databases to report diagnoses, classifying morbidity data for indexing, medical care review, and capturing basic health statistics. Comparing both versions, ICD‐9 diagnosis codes are generally broad, while ICD‐10 are more specific and contain much more detail, which will allow better assessment of the quality, safety, efficacy, and research of healthcare data. ICD classification is an integral part of most information systems, for both printed and paperless forms.

Potential errors in the usage of ICD codes lie more with their specificity, which is a result of formulation and designing based on disease nomenclature,^75^, ^76^ misdiagnosis,^77^ and documentation in electronic and paper records.^78^ The degree to which the severity of these consequences is realized depends on the education of medical professionals and availability of tools to increase the awareness on code applicability and potential discrepancy sources.^79^ In the United States, the FDA maintains NDC for uniquely labeling and identifying commercially available drug products. NDC is composed of FDA‐assigned code labels: product codes to identify specific drug strength, dosage strength, and formulation; and package codes to categorize package sizes and types. These codes are well adopted in the United States, especially among pharmaceutical manufacturers, wholesalers, healthcare institutions, Medicaid, Medicare, and managed care organizations for inventory control, identification of potential drug‐drug interactions, medical precautions and drug claims, utilization, review, physician order entry, and clinical patient profile screening and counseling. Like ICD codes, NDC codes have also been inspected for quality and comprehensiveness by the FDA in the United States.^80^, ^81^ Most medical professionals have to memorize these healthcare codes or consult a reference book or a website when needed. For medical users, to fully benefit from ICD and NDC classifications, a user‐friendly comprehensive database with associated details is needed.

Our focus is to create database tool that provides efficient access to clinical disease and drug classification codes, considered as one of the foundations for clinical operations and research using medical records. We have developed an integrated, comprehensive, and centralized database enabling efficient management and access to the most recent versions of ICD‐9, ICD‐10, and NDC for epidemiology, health management, clinical purposes, and scientific research. This mobile module assists personnel especially from clinical, pharmaceutical, and life science communities using medical databases to validate their data, build upon classified code lists, match disease definitions, share clinical data as research objects, and produce study replications. As most of the medical codes and terminologies are difficult to understand and link to genomics data, our social pledge is to educate individuals by providing them with an app to query, easily explore, and gain information on the classification of signs, symptoms, diagnoses, medications prescribed by healthcare institutions, and genes and SNPs.

PAS can prove to be a fundamental clinical decision support system application by making the “clinical language” as meaningful and accurate for system communication with complete information exchange. Inaccurate information can compromise the integrity of the clinical content that is transmitted from one setting to another whether it is between providers, EMTs, ambulances, clinics, hospitals, and other settings of care. This may be the only information available for clinicians to rely on at the point of care. Developing and implementing a centralized mobile accessed repository with access to both NDC and ICD, can assist healthcare providers, researchers, and pharmaceutical companies to integrate their health information systems interorganizationally, exchange product‐specific drug information, establish drug dispensing among pharmacy communities, develop clinical decision‐support systems for disease state management, provide point‐of‐care drug information and patient counseling services, determine the validity of research, and perform effective comparisons between studies.

### 2.2 Genomics database development of genes and variants

Currently, challenges in synthesizing the existing data resources are due to lack of technical standards for exchange and reporting of actionable genetic variants and associated phenotype, thus limiting the interoperability.^1^ Many complex diseases, such as cancer, develop with the sporadic acquisition of genome alterations. These alterations may cause functional cellular issues or be inert. Not all mutations contribute equally to the cancer type in which they are found. Although the number of unique variants for each cancer genome can be very high, only a few variants will be critical for the development of the tumor. This necessitates the need of bioinformatics tools linked to comprehensive knowledge bases for identifying genetic variants for potential clinical action. In this part of the study, we looked at the existing resources that provide disease‐variants information and then designed and developed a new database based on the collection of curated distinct and actionable genes data from available genomics sources worldwide, including CNVD,^82^ Ensembl,^83^ GenCode,^84^ ClinVar, GeneCards, DISEASES, HGMD, OMIM, GTR, Orphanet, Novoseek (http://www.novoseek.com), Swiss‐Prot, and LncRNADisease. Disease Ontology provides information about human disease ontologies; DiseaseEnhancer offers disease‐associated enhancers; DISEASES delivers disease‐gene associations, cancer mutations, and GWAS‐based SNPs; DisGeNET is the catalogue of genes and variants, associated with human diseases; eDGAR provides information about disease‐gene associations with annotated relationships; GTR shares information about genetic‐based disorders; MalaCard is the collection of gene‐disease associations; SwissVar groups single amino acid polymorphisms; miR2Disease offers information about human diseases involving microRNA deregulation; HGMD is the collection of published germline mutations in genes associated with human inherited diseases; ClinVar offers information about single amino acid polymorphisms; Orphanet provides information about rare diseases; and Gene2Function maps orthologs among human genes and common genetic model species (Table 4).^1^

**TABLE 4:** Gene‐disease databases comparison based on the following features: gene to disease, disease to ICD, data types, data sources, gene capacity, latest update, search results, and user friendly interface

Database	Weblink	Gene to disease	Disease to ICD	Data types	Data sources	Gene capacity	Latest update	Search results	User friendly	Last date accessed
Disease Ontology	http://disease-ontology.org/	Yes	No	Human disease ontology	MeSH; ICD; NCI's thesaurus; SNOMED and OMIM	Not found	2018	DOID; Disease name	Yes	01‐22‐2020
DiseaseEnhancer	http://biocc.hrbmu.edu.cn/DiseaseEnhancer/	Yes	No	Disease‐associated enhancers	1866 publications	308 genes	2018	ID;Chr; Position; Disease; Target Gene	Yes	01‐22‐2020
DISEASES	https://diseases.jensenlab.org/Search	Yes	Yes	Disease‐gene associations; cancer mutations; genome‐wide association studies	GHR; UniProtKB; GWAS results from DistiLD and COSMIC	17 606 genes	2018	Genes; Identifiers; Disease name; Scores; Confidencity; Publication	No	01‐22‐2020
DisGeNET	http://www.disgenet.org/web/DisGeNET/menu	Yes	No	Genotype‐phenotype relationships	CTD; UniProt; ClinVar; Orphanet; RGD; MGD; GAD	17 381 genes	2018	Gene; Disease; Disease Class; Semantic Type; PMIDs; SNPs	No	01‐22‐2020
eDGAR	http://edgar.biocomp.unibo.it/gene_disease_db/	Yes	No	Gene/disease relationships	OMIM; Humsavar; ClinVa	3658 genes	2016	Gene; Number of associated diseases; Associated diseases identifiers; Associated diseases names	Yes	01‐22‐2020
GTR	https://www.ncbi.nlm.nih.gov/gtr/	Yes	No	Genetic test information by providers	ClinVar; Genetics & Medicine; GeneReviews; MedGen; OMIM; Orphanet; NHGRI Glossary…	16 451 genes	2018	Associated conditions; Copy number response; Genomic context; Variation; Related articles in PubMed	Yes	01‐22‐2020
MalaCard	https://www.malacards.org/	Yes	Yes	Gene; genomics; proteins; gene ontology; pathways; drugs; diseases	76 various sources https://www.malacards.org/pages/info#whats_in_a_malacard	71 150 genes	2018	Symbol; Aliases; Disorder; Score; IsCancerCensus; Sources	Yes	01‐22‐2020
SwissVar	https://swissvar.expasy.org/	Yes	No	Single amino acid polymorphisms (SAPs) and diseases	Disease:UniProtKB/SwissProt and their mapping to MeSH terms; variant:ModSNP database	Not found	Not found	Accession; Disease; Variant	Yes	01‐22‐2020
miR2Disease	http://watson.compbio.iupui.edu:8080/miR2Disease/index.jsp	Yes	No	MicroRNA deregulation in human diseases	Researchers submission; Pubmed text‐mining	349 miRNAs	2008	miRNA; Disease; Relationship type; Target Gene; Reference	No	01‐22‐2020
HGMD	http://www.hgmd.cf.ac.uk/ac/index.php	Yes	No	Germline mutations	GDB; OMIM; 250 journals	6662 genes	2017	Disease/phenotype; Number of mutations; Gene Symbol	Yes	01‐22‐2020
ClinVar	https://www.ncbi.nlm.nih.gov/clinvar/	Yes	No	Relationships among variations and human disorders	Clinical testing; research; extraction from the literature	26 000 genes	2018	Variation; Genes; Conditions; Clinical significance; Review status	Yes	01‐22‐2020
Orphanet	https://www.orpha.net/consor/cgi-bin/index.php	Yes	Yes	Rare diseases and orphan drugs	OMIM; ICD10; MeSH; MedDRA; GARD and UMLS	15 470 genes	2018	Genes; Disease; ORPHA ID; ICD	No	01‐22‐2020
Gene2Function	http://www.gene2function.org/search/	Yes	No	Orthologs among human genes and common genetic model species	OMIM; EBI; GWAS; HGNC; MOD; DIOPT; GO; SGD; ORF; NCBI; PDB; Uniprot	10 499 genes	2017	Gene ID; Gene Symbol; Human Disease; Species Name…	No	01‐22‐2020

Scientists around the world have performed large‐scale GWAS^23^ based on the simple idea that if a genetic variant increases disease risk, it should be more frequent among disease cases than healthy controls. Their breakthrough study initially revealed 24 significant disease‐associated DNA variants.^23^ The richness of genetic variations in the human genome has been further corroborated by the several whole genome‐sequencing studies, revealing plenty of new SNPs, insertions and deletions (indels), copy number variants, and other structural variations. Later, several thousands of GWAS have produced tens of thousands of strong associations between genetic variants and one or more complex traits.^23^ GWAS require high marker density or resolution, in which several hundred thousands of SNPs are needed spanning the whole genome, to achieve comprehensive coverage and adequate statistical power to detect unknown disease variants through linkage disequilibrium.^23^, ^85^, ^86^ Most of the SNPs are predicted to be neutral without functional effects and due to their abundance in the human genome, SNPs have become useful genetic markers in GWAS. The proportion of mutations causally implicated in cancer is still unknown especially due to the high number of variations between different tumors.^66^ Patient stratification into subpopulations based on their genetic risk factors will allow us to understand the role the environment plays in triggering disease. Ultimately, comprehensive answers will require much larger patient populations, detailed clinical databases, and sophisticated technical approaches to translate GWAS findings into medical practice.

Further extending the database, we added collection of germline and somatic mutations released globally, including GWAS Catalog (data mapped to genome assembly), COSMIC, CNVD, SwissVar (https://swissvar.expasy.org), and so on. The criteria for selecting variant databases included classification of germline and somatic mutations and their associations with diseases. We were interested in finding mutation databases, which report all available and actionable genes with germline and/or somatic mutations with direct or indirect association with classified diseases and their ICD codes. Due to the heterogeneous, complex, and high volume, variant data evaluation and extraction process was time‐consuming and laborious. It took us months to download, classify, upload, and integrate data in to SQL servers. For annotation, we integrated mutations with their respective genes, and linked that information to related diseases. We performed extensive data quality assessment to ensure authentic, up‐to‐date and standardized information. Our database is freely available public repository offering annotated disease, drug and genomics data by integrating genes, SNPs, diseases, and drugs.

Our study intended to correlate the genotype with disease phenotype, and to identify all the genetic variations that are associated with the diseases. The objective was to create broad database, which will help uncover common DNA differences among people who influence traits, functions,^87^ or raise the disease risk.^88^ Our comprehensive approach provides access to the information related with biological roots of common and rare diseases to facilitate probing for anonymous disease variants.^89^, ^90^ Our designed database has the potential to extend and support integration of genomic information into EHR systems defining the population frequency of variants by assisting curation of massive population biobanks.^91^, ^92^ Some studies have benefitted from identifying high‐risk patients and crafting precision medicine therapies for complex diseases (cancer),^93^, ^94^, ^95^ metabolic diseases (type 2 diabetes), autoimmune diseases (psoriasis), and psychiatric diseases (schizophrenia)^96^ by discovering gene variants associated with disease development. The outcome of these revolutionizing studies raises the fact that well structuring and packaging of the gene‐variant data could pave way for discovering the genetic roots of common heritable disorders, more accurate identification of people at risk, better prevention strategies, and more effective therapies with fewer adverse effects. This is where our app comes as a complement to the utilization of genomics and healthcare data in refining analytical approaches and pursuing functional role of rare variants.

We open up the world of somatic variations in cancer as they are used in clinical studies and molecular pathology to characterize tumor types, to improve the best suited treatment choice, and to predict response to treatment.^97^, ^98^ In a clinical setting, these discoveries are named “secondary findings,” or “secondary variants (SVs)” and have to be distinguished from “incidental findings” that are found in the genes linked to the tested disease.^99^ This requires extra workload needed for variant interpretation and confirmation.^100^, ^101^ For this reason, we address the needs of clinical research as it provides a great resource database of published gene variants, which can be easily integrated into any system to filter out candidates for the discovery of causal variants. This can aid the clinical genetics community to form expert panels, to perform high‐level curation for variant interpretations for clinical significance and identifying genetic variants for yielding new therapeutic targets and biomarkers for antibodies and vaccines.

### 2.3 iOS app development for mobile access to annotated information

**FIGURE 1:**
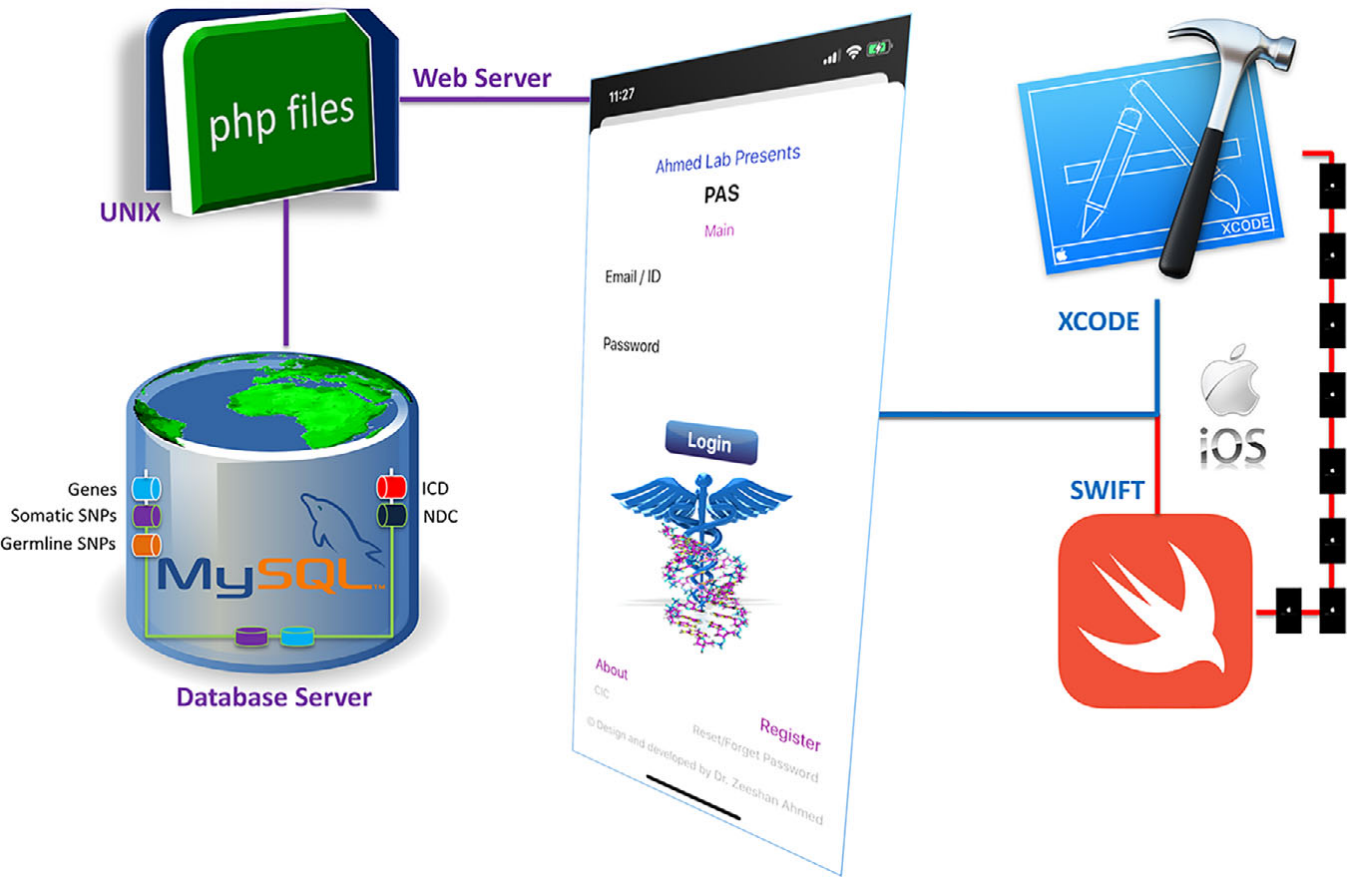
PAS components design, development, and data flow. PAS is an iOS app developed with Swift programming language, XCODE integrated development environment for MacOS, MySQL database management system, PHP scripting language, and UNIX‐based web and database servers

With the growing trend of adapting smart phone applications into biomedical research, it will be helpful to develop comprehensive, integrated, and searchable mobile database for genes, variants, and related disease and drugs. We have developed an iOS app with Swift programming language, using the XCODE integrated development environment for MacOS (Figure 1). Its database is modeled and hosted within the MySQL database management system, deployed with in a highly secured environment. The secure socket layer (SSL), otherwise known as transport layer security, is implemented for authentication purposes, and data integrity and confidentiality. PAS is based on mobile interface linked to a dedicated database server. Database is not the part of app but can be accessed via web server. Dynamic web‐based modules are developed using the PHP scripting language to facilitate data migration between the iOS app and MySQL database server. One of the most difficult and complex tasks of implementing an iOS app connecting a mobile interface via PHP programmed modules to an external web‐based MySQL server for data exchange is the integration of all modules developed using different programming languages and processed through different compilers/interpreters that sometimes cause logical errors, which are hard to debug. Using a secure key, SSL establishes an encrypted link between web services and the mobile app.

We established efficient machine‐to‐machine and human‐to‐machine connectivity‐based networked communication. We designed five‐layered architecture, based on perception, processing, business, application, and network layers. Perception is the physical layer with graphical user interface to get user input and deliver output (Supplementary Material: PAS Guide and Workflow Design). Processing is the middleware layer, responsible for handling user requests. Business layer provides overall IoT system actions and functionality, forwards processed user requests to the database server via web server, and brings the results to the user interface. Application layer delivers application‐specific services to the user. Network layer is responsible for receiving data from the perception layer and transmitting it to the application layer through available network technologies, and support data management from storing to processing with the help of middleware. The rapid growth in genomics data sources and types requires robust and automatic translation to different formats. One of the greatest challenges here was to deal with the daunting complexity of the genomics data, as the diverse structures of its databases and exported files are with different numbers of fields and data types. We developed a data extraction, transfer and loader (ETL) software in Java, which adopts supervised learning method to understand and validate structure of data source. In our case, data were available in different file formats (eg, csv, excel file, text, etc), which were uploaded using newly developed ETL software. We run ETL process for weeks to upload the data, especially somatic mutations, as the numbers were in millions.

Developed app is tested using XCODE‐provided simulator and real Apple mobile devices with the most recent iOS versions, and is reviewed and approved by the Apple, and is freely available to download at the App Store. The graphical user interface of PAS is very flexible and can be installed and well executed at both iPhone and iPad devices. Furthermore, it facilitates automatic vertical and horizontal resolution configuration. Its technology benefits include enhanced security, filtered audience, better user experience, flexible user interface, stable movement and orientation, internet access, and consistent system environment. While development, app was divided into three different modules: clinics, genomics, and clinical genomics. Product line architecture of app is based on the *Butterfly* model,^102^, ^103^ where all major modules are capable of performing individual key roles and can integrate with each other. The overall design and workflow is flexible to accommodate new releases and updates of genes, SNPs, diseases, and drugs without requiring its users to install its new version but automatically updating database. Having updates at the user interface, automatics upgrade notifications will be served to the user for downloading latest released version of the app. Gene module is designed to simplify navigation across the landscape of gene annotation resources by an efficient mobile record search engine, which is based on standardized genes and related diseases to help explore multipurpose clinical and genomics concepts in meaningful ways. Germline and somatic SNP modules are composed of mutations reported for genes and their combinations with disease traits, when disease and drug modules are based on ICD‐9, ICD‐10, and NDC. The normalized relational database includes lists of WHO‐maintained ICD‐9 and ICD‐10 lists downloaded from the Center for Medicare & Medicaid Services, and FDA‐approved NDC codes.

**FIGURE 2:**
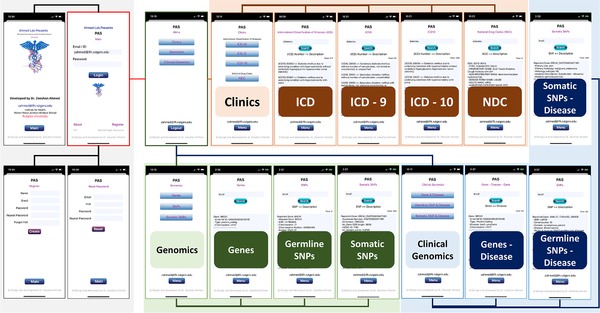
PAS graphical user interface and work flow design

The graphical user interface implements human‐computer interaction principles (Figure 2). It provides user profile, login, and password management modules, which requires new users to first create an account and login with valid credentials. The major reasons to request users to first register and login is to apply security features to the app to track its usage and backtrack in case of any trouble, breach, and violation. In the future, we are looking forward to implement AI and machine learning‐based features to help users in finding data of their interest based on their search history, and having their profile will be extremely useful in such cases. Moreover, having users email address is useful, especially to inform for major updates to the app and database. At successful login, users are directed to the main menu leading to the clinics, genomics, and clinical genomics interfaces. Genomics leads to three subinterfaces: genes, SNPs, and somatic SNPs. Genes allow users to search for genes and relational information, which includes gene name, Ensembl ID, type, and chromosome. SNPs allow users to search for SNP and relational information, which includes reported gene, mapped gene, SNP ID, chromosome, chromosome position, region, context, platform, and PUBMED ID. Somatic SNPs allow users to search for somatic mutations for a gene with detailed gene, mutation, and sample information, which includes reported gene name, accession number, ID tumor, gene coding sequences (CDS)^104^ length, sample name, ID sample, primary site, mutation ID, Site subtype, mutation, mutation description, mutation CDS, Genome Reference Consortium Human (GRCH), mutation genome position, functional analysis through hidden Markov models (FATHMM) prediction,^105^ FATHMM score, mutation somatic status, sample type, tumor origin, ID study, age, and PUBMED ID for the published study.

Clinical genomics leads to three subinterfaces: genes & disease, germline SNP & disease, and somatic SNP & disease. The gene to disease interface let users search for genes and related diseases, and vice versa. SNP to disease interface let users search for related diseases, which includes reported gene, SNP, chromosome, context, disease, and study. The somatic SNP & disease interface gives concise information on gene search, which includes reported gene name, primary site, primary histology, histology subtype, mutation ID, mutation description, mutation genome position, and accession number. Clinic leads to four subinterfaces: ICD‐All, ICD‐9, ICD‐10, and NDC. ICD‐All allow users to search for ICD‐9, ICD‐10, or disease‐related information for both ICD‐9 and ICD‐10 databases. The ICD‐9 interface let users search only ICD‐9 and similarly, the ICD‐10 interface only searches for ICD‐10 code lists and related details. The NDC interface enables users to search for NDC and related details, which includes product ID, product NDC, product type name, proprietary name, nonproprietary name, dosage form name, route name, marketing category name, application number, labeler name, substance name, active numerator strength, active Ingred unit, pharm classes, and listing records certified through.

## 3 RESULTS

PAS is an easy‐to‐use application designed to simplify navigation across the landscape of gene annotation resources by an efficient mobile record search engine, which is based on standardized genes and related diseases to help explore multipurpose clinical and genomics concepts in meaningful ways. Here, we present reproducible results to help users better understand the data mining, management, search, and analytics capabilities of all four modular apps and integrative‐databases of PAS.

**FIGURE 3:**
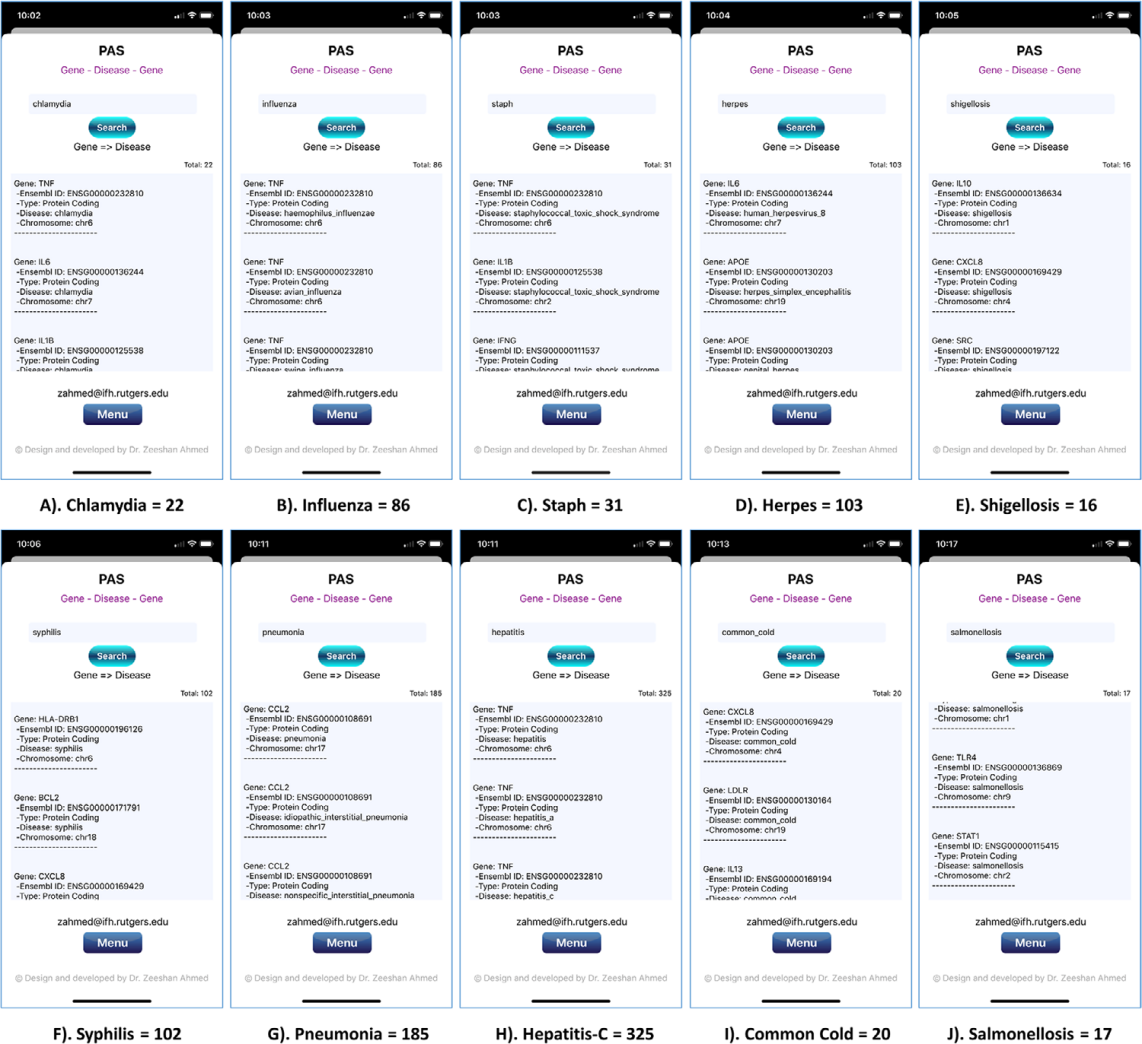
PAS (iPhone 11 Pro) screenshot of gene results from searches for the 10 most common infectious diseases in the United States: (A) 22 chlamydia, (B) 86 influenza, (C) 31 staph, (D) 103 herpes, (E) 16 shigellosis, (F) 102 syphilis, (G) 185 pneumonia, (H) 325 hepatitis‐C, (I) 20 common cold, and (J) 17 salmonellosis

Validating PAS (Figure 3), we designed use case for 10 most common infectious diseases, which includes gene results with 22 chlamydia (sexually transmitted infection caused by the bacterium *Chlamydia trachomatis*)^106^ (Figure 3A), 86 influenza (viral infection that effects respiratory system)^107^ (Figure 3B), 31 staph (infection caused by the Staphylococcus genus of bacteria)^108^ (Figure 3C), 103 herpes (infection caused by *Herpes Simplex Virus*)^109^ (Figure 3D), 16 shigellosis (diarrheal disease caused by a group of bacteria called *Shigella*)^110^ (Figure 3E), 102 syphilis (sexually transmitted infection caused by the bacterium *Treponema pallidum*)^111^ (Figure 3F), 185 pneumonia (infection of the lungs)^112^ (Figure 3G), 325 hepatitis‐C (viral infection that causes liver inflammation)^113^ (Figure 3H), 20 common cold (viral infection of your nose and throat)^114^ (Figure 3I), and 17 salmonellosis (bacterial disease that affects the intestinal tract)^115^ (Figure 3J).

**FIGURE 4:**
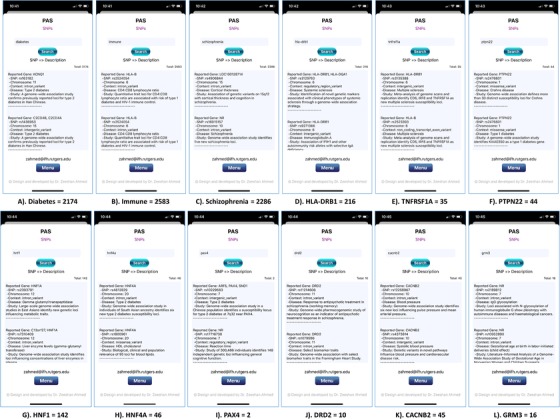
PAS (iPhone 11 Pro) screen shots present searched results for all possible SNPs related to diabetes (2174), immune (2583), and schizophrenia (2286) diseases; searched results for diabetes‐related SNPs: HNF1 (142), HNF4A (46), and PAX4 (2); searched results for auto immune disease‐related SNPs: HLA‐DRB1 (216), TNFRSF1A (35), and PTPN22 (44); and searched results for schizophrenia‐related SNPs: DRD2 (10), CACNB2 (45), and GRM3 (16)

Validating PAS (Figure 4), we designed two use cases based on three different most common genetic diseases: diabetes (group of metabolic disorders characterized by a high blood sugar level),^116^ schizophrenia (serious mental disorder),^117^ and autoimmune diseases (condition arising from an abnormal immune response to a normal body part).^118^ In first use case: 2174 results are obtained while looking for all available SNPs related to the diabetes (Figure 4A), 2583 results for autoimmune disease (Figure 4B), and 2286 results for schizophrenia (Figure 4C). Obtained results also include information about reported genes, chromosomes, contexts, diseases, and studies. In second use case: we randomly selected three SNPs mainly lined to autoimmune diseases, diabetes, and schizophrenia, and looked for all possible associations with major and other diseases. SNPs for autoimmune diseases include major histocompatibility complex, class II, DR Beta 1 gene HLA‐DRB166 (mainly associated with rheumatoid arthritis), tumor necrosis factor receptor type 1 gene (TNFRSF1A)^95^ (mainly associated with multiple sclerosis and ankylosing spondylitis), and protein tyrosine phosphatase nonreceptor type 22 gene (PTPN22)^29^ (mainly associated with increased risk of type 1 diabetes, systemic lupus erythematosus, vitiligo,^119^ autoimmune thyroid disorder,^120^ and ulcerative colitis^121^ but is protective against Crohn's disease). Obtained results present 216 SNP‐disease combinations for the HLA‐DRB1 (Figure 4D), 35 for the TNFRSF1A (Figure 4E), and 44 for the PTPN22 (Figure 4F). SNPs for diabetes include hepatocyte nuclear factor 1 gene (HNF1),^94^ hepatocyte nuclear factor 4‐alpha gene (HNF4A)^94^ (involved in monogenic conditions) and paired box 4 gene (PAX4)^94^ (identified in East Asian population). Obtained results include 142 SNP‐disease associations for the HNF1 (Figure 4G), 46 for the HNF4A (Figure 4H), and only 2 for PAX4 (Figure 4I). SNPs for schizophrenia include dopamine receptor D2 gene (DRD2), calcium voltage‐gated channel auxiliary subunit beta 2 gene (CACNB2), and glutamate metabotropic receptor 3 gene (GRM.3)^40^ Obtained results present 10 SNP‐disease combinations related to the DRD2 (Figure 4J), 45 related to the CACNB2 (Figure 4K), and 16 related to the GRM3 (Figure 4L).

**FIGURE 5:**
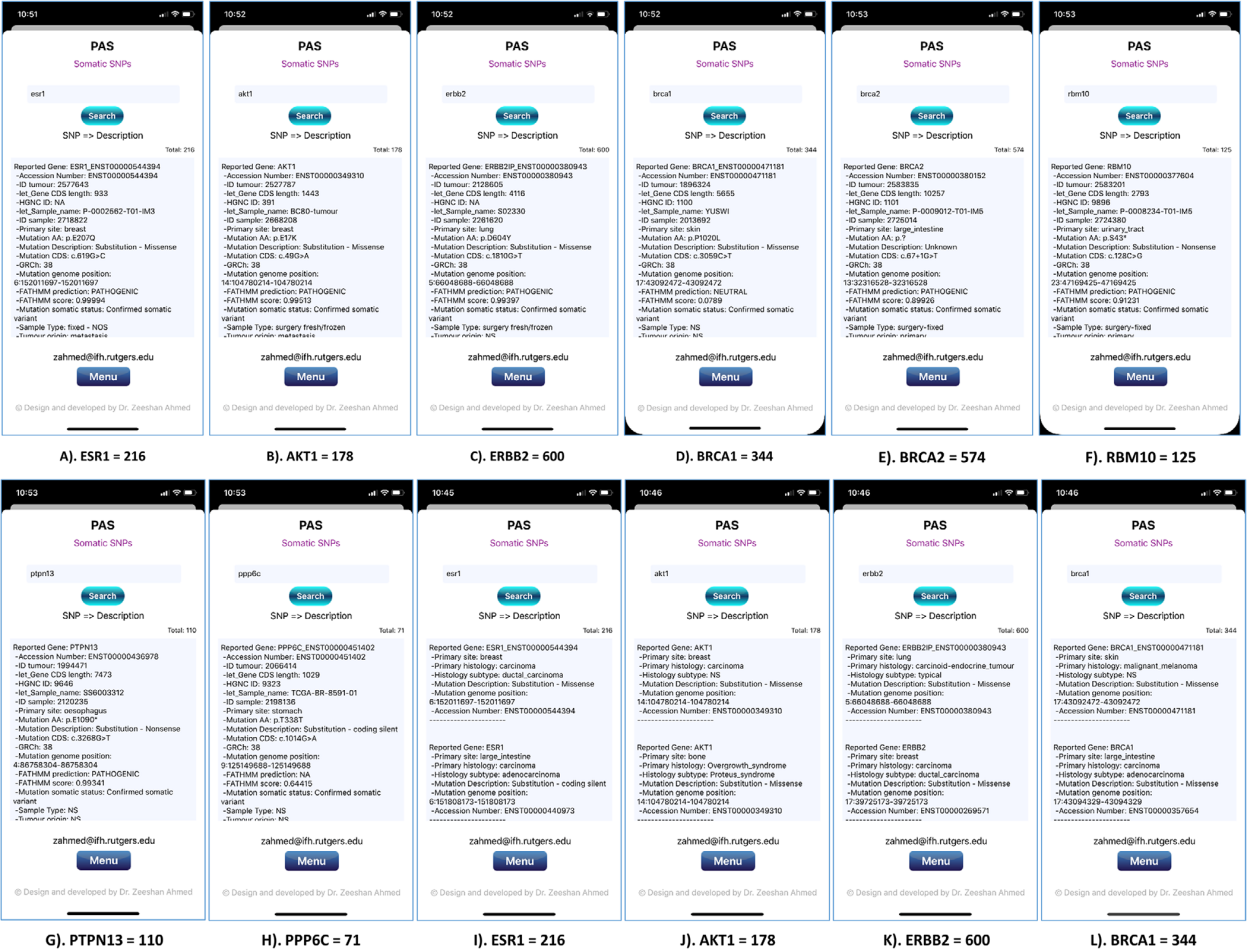
PAS (iPhone 11 Pro) screen shots present eight searched results for all possible SNPs related to the eight different genes: 216 results for “ESR1” (A), 178 results for “AKT1” (B), 600 results for “ERBB24” (C), 344 results for “BRCA1” (D), 574 results for “BRCA2” (E), 125 results for “RBM10” (F), 110 results for “PTPN13” (G), and 71 results for “PPP6C” (H). Figure 4 also presents four examples of searched results for all possible SNPs and their diseases relationships: 216 results for entered and searched keyword “ESR1” (I), 178 results for entered and searched keyword “AKT1” (J), 600 results for “ERBB24” (K), and 344 results for “BRCA1” (L)

We designed another use case to validate PAS for gene‐variants (Figure 5), which include somatic mutations (hallmark for cancer) related to eight different reported genes: estrogen receptor 1 gene (ESR1),^128^ AKT serine/threonine kinase 1 gene (AKT1),^129^ Erb‐B2 receptor tyrosine kinase 2 gene (ERBB2), breast cancer type 1 susceptibility protein gene (BRCA1),^5^ breast cancer type 2 susceptibility protein gene (BRCA2),^5^ RNA binding motif protein 10 gene (RBM10),^99^ protein tyrosine phosphatase nonreceptor type 13 gene (PTPN13), and protein phosphatase 6 catalytic subunit gene (PPP6C).^93^ Obtained results present 216 combinations of mutations and related diseases for “ESR1” (Figure 5A and I), 178 for “AKT1” (Figure 5B and J), 600 for “ERBB2” (Figure 5C and K), 344 for “BRCA1” (Figure 5D and L), 574 for “BRCA2” (Figure 5E), 125 for “RBM10” (Figure 5F), 110 for “PTPN13” (Figure 5G), and 71 matches for “PPP6C” (Figure 5H).

**FIGURE 6:**
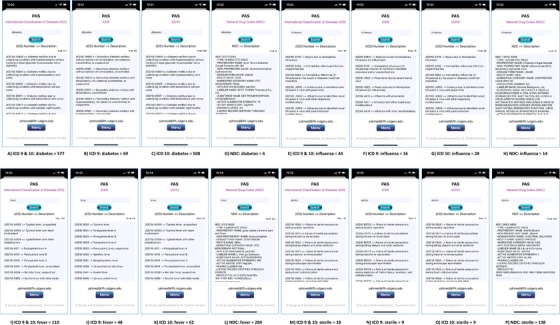
PAS (iPhone 11 Pro) screen shots present searched results obtained during use cases for four different diseases: diabetes, influenza, fever, and sterile. Diabetes includes total 577 ICD (A), 69 ICD9 (B), 508 ICD10 (C), and 6 NDC (D). Influenza includes total 44 ICD (E), 16 ICD9 (F), 28 ICD10 (G), and 14 NDC (H). Fever includes total 110 ICD (I), 48 ICD9 (J), 62 ICD10 (K), and 284 NDC (L). Sterile includes total 18 ICD (M), 9 ICD9 (N), 9 ICD10 (O), and 138 NDC (P)

Validating PAS for disease codes (Figure 6), we designed four use cases based on four different most common diseases: diabetes, influenza, fever, and sterile. In all use cases, we looked for available codes (ICD, ICD9, ICD10, and NDC) related to these four diseases. Obtained results for diabetes include total 577 ICD (Figure 6A), 69 ICD9 (Figure 6B), 508 ICD10 (Figure 6C), and 6 NDC (Figure 6D) codes. Obtained results for influenza include total 44 ICD (Figure 6E), 16 ICD9 (Figure 6F), 28 ICD10 (Figure 6G), and 14 NDC (Figure 6H) results. While looking for fever, we found total 110 ICD (Figure 6I), 48 ICD9 (Figure 6J), 62 ICD10 (Figure 6K), and 284 NDC (Figure 6L) results, and obtained total 18 ICD (Figure 6M), 9 ICD9 (Figure 6N), 9 ICD10 (Figure 6O), and 138 NDC (Figure 6P) results for sterile. High resolution images of figures (3, 4, 5 and 6) are attached in supplementary material.

**FIGURE 7:**
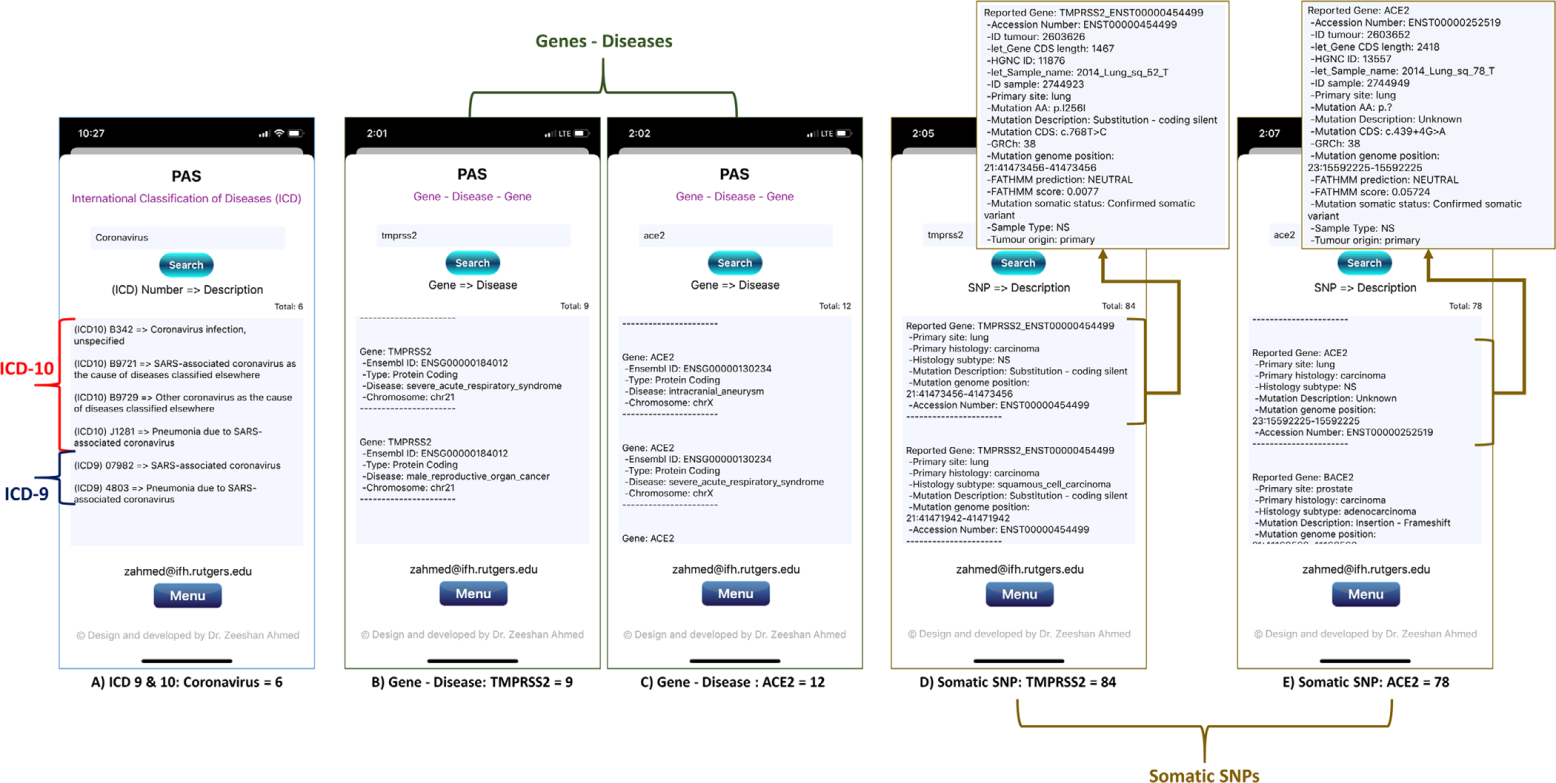
PAS (iPhone 11 Pro) screen shots present searched results obtained for infectious disease i.e., Coronavirus (Fig. 7A), genes (Fig. 7B and 7C), and variants (Fig. 7D and 7E) including ACE2 and TMPRSS2

Since last few months, an infectious disease i.e., Coronavirus (COVID‐19) caused by the SARS‐CoV‐2^130^ has effected the whole world. Despite many significant scientific and medical discoveries, the genetics of pandemic COVID‐19 remains far from clear. As of today (04/16/2020) over 657 720 cases have been confirmed and over 33 460 deaths have been reported in USA as a result of COVID‐19, when over 2 101 164 people have been effected and over 140 773 have died worldwide. However, over 53 322 people have recovered in USA, and over 532 830 globally (https://google.com/covid19‐map/?hl=en). Highlighting the useful contribution of our app and database in this situation, users can look for the relevant disease (Coronavirus) specific information (Fig. 7A), genes (Fig. 7B and 7C), and variants (Fig. 7D and 7E) including ACE2 and TMPRSS2.^131^

PAS brings the power of quicker excess to mutation data in healthcare, especially cancer research as an iOS app and a unique resource as a cancer‐variant database. Over 450 research articles are indexed on PubMed, and only ∼5% of those are supplemented with clinical codes,^72^ and few for NDC. Drawing on patient‐based classified clinical entities of interest often requires iterative code‐based searches in medical databases and help from clinical experts. The resultant information could lead to a particular disease condition as a combination of codes representing diagnoses, symptoms, prescribed drugs, and diagnostic tests, as well as a simple code or list. In such cases, our app aims to assist clinicians and researchers with easy navigation and free portable access to diagnostic and drug codes for efficient and quick disease and drug classifications. Individual practices can also benefit from PAS with better metrics to measure their performance relative to their peers, contain costs, spot disease risks, identify patients in need of immediate disease management, and uncover opportunities for greater efficiency. This will enable physicians to funnel the right patients into the right programs, and better refine disease management for those already in a program, outreach to the patients to deliver preventative care versus more serious and costly care when a condition becomes an emergency. The comparability factor for quality measure reporting is of special interest to organizations, while patients may want to look up the medical billing codes. The codes may be of interest to the medical biller who can send the coded claim to the health insurance company for processing. Researchers may use the data to determine disease incidence and prevalence across geographic areas, ages, or in conjunction with other diseases.

## 4 DISCUSSION

The pursuit of the genetic roots of common illnesses is, at its heart, a quest for better prevention and treatment of disease. We are entering the era of personalized medicine in which an individual's genetic makeup will eventually determine how a doctor can tailor his or her therapy. Therefore, it is becoming critical to understand the genetic basis of common diseases, for example, which genes predispose individuals to diseases, which gene interactions affect disease risk, and how do rare genetic variants contribute to diseases? Human diseases are at the heart of extensive research encompassing genomics, bioinformatics, systems biology, and systems medicine. To get new insights into disease taxonomy, etiology, and pathogenesis, it is important to understand how diseases are related to each other.^42^ In the past, various efforts have been made in deciphering diseases to facilitate predictive diagnosis and thereby guide treatment factors, which include drawing disease relationships using clinical manifestations,^118^ healthcare records, images and data generated using wearable technology and AI,^50^ along with information encapsulated within related genes,^122^ signaling,^123^ and metabolic pathways,^124^, ^125^, ^126^ microRNAs,^127^ chemo‐centric views,^128^ phenotypic characteristics, microbes,^129^ and proteins.^132^ Differences among humans can be divided into two broad categories: biological and environmental. Variation is inherent in humans and it is the result of fundamental biological and environmental processes, ensuring vitality, ability to adapt to changing environments and even the very survival. The former includes genetic mutations, while the environment induces somatic mutations. A typical gene can have a multitude of variants that have not yet been documented to have a relationship with a disease or a phenotype. Gene‐disease data are highly significant at every level of biological research and healthcare but with inconsistencies and inabilities in terms of gene annotation, specificity of disease classification terminologies adds to the complexity and lack of an efficient integrative searchable system that makes it difficult to comprehend the underlying implications.

Genome‐scale (genome, transcriptome, proteome, metabolome, microbiome, and epigenome) science is becoming increasingly common with the advancement of high‐throughput technologies. The technological developments have facilitated and accelerated the process of identifying genetic variations, especially with the arrival of NGS technologies, which have made whole genome sequencing and the 1000 Genomes Project feasible.^133^, ^134^ A key challenge in this realm is NGS interpretation. Scientists are faced with the daunting challenge of identifying candidate genes that are relevant to their biological system of interest. Most often, the researcher only has direct knowledge of a few, if any, candidate genes. The clinical interpretation of the significance of specific gene variants can be unique to a patient. Variability in interpretation for sequence variants is due, in part, to the lack of standard curated information to support clinical decision making.^1^ Currently, the investigation of existing databases is required to assess the potential significance of even one sequence variant, and that is a cumbersome, time‐consuming, and increasingly unfeasible process with regard to the identification and reports of variants in actionable genes because of the absence of a standard centralized platform for connecting genes to their disease phenotypes.^135^ In this study, we present our research to collect, explore, and share genes and disease information to support pathology and epidemiology for the implementation of precision medicine. It is founded on clinical, scientific, and modern technologies posit to support healthcare through free portable public research enabling scientific data retrieval using efficient mobile‐based tools.

One platform that has proven to be an efficient tool in several areas, including healthcare, is the mobile application. Since the release of the first iPhone in 2007, Apple has sold more than a billion iPhones worldwide. As iOS systems have become increasingly popular and the medical community is flocking to iPhones and iPads, there is no iOS app publically available, which can provide unified access to genomic, clinical, and pharma databases with easy navigation and free portable access to genes and related diseases and drugs for efficient and robust classifications. The reasons could be extensive heterogeneity of clinical and genomic data collection and management, or addressing complexities of implementing an Apple mobile app. The objective of our research is to create centralized gene‐SNP‐disease‐drug database, which not only stores, organizes, and shares data in a structured and searchable manner but also facilitates data retrieval with smartphone application, including visualization features for analytics and implementing all available and actionable genes‐based data classification. Looking at the existing gaps and unmet clinical, research and market needs of healthcare, genomics, pharmaceutical, and biomedical communities, we offer a new solution with a social pledge to help individuals by providing them with an interactive app to query, easily explore, and access information on gene annotation and classified disease phenotype with greater visibility and easy browsing. The gene‐SNP‐disease‐drug querying ability offered by the app provides the user with an important knowledge discovery tool, just a click away from any location.

Employing the power of mobile technology to integrate multiple data streams is certainly challenging, since it involves a huge amount of data collection, structuring, management, processing, authentication, integration, and sharing. However, it is tremendously rewarding to assist the clinicians to directly interpret a patient's genomic profile and collaborate with the scientists to translate variant data to therapy. The genetic architecture of complex diseases remains elusive. It is unclear how much each type of genetic variation contributes to inherited risk and the relative proportion of rare versus common variants. When collecting the data sources, along with the last maintenance update year, it was noticed that some databases have not been updated in the last few years and some have very limited data (eg, over a few hundred or a thousand genes).^1^ Most databases and tools have multiple sources at backend, which at times prove to be a double‐edged sword. On the one hand, more data sources enrich the capacity of the databases and on the other hand, too many data sources may make it difficult to avoid entry of uncertain or erroneous data. We tried to address this data heterogeneity by standardizing the data on a single platform.

Mapping genes to their diseases and at the same time, mapping the diseases to their respective codes will provide a crosscut to find drugs and the therapeutics for specific patients when our database is applied to real human medical records linking their disease diagnosis to known and identified causative genes and variants. The underlying assumption here is that creating a database with smart distillation and abundant distribution of genes and SNPs linked to the classified diseases and drugs through their description and IDs (eg, ICD and NDC) can support both clinical and research environments.^1^ We have performed and reported (Table 4) comparison of different gene‐disease databases. We evaluated, whether these provide information related to genes, gene to disease, disease to ICD code, data types, data sources, gene capacity, latest updates, searched results, and user friendliness. There are only few databases available (eg, Disease Ontology, DISEASES, and Orphanet), which provide ICD codes for each disease but not directly linked to the genes. Such database must not be redundant and should only include human reference genome and disease‐based information collected from valid sources available worldwide. It is very important to facilitate interested users with efficient, user friendly, easy navigation, and free portable access to the database using platforms that have proven to be efficient tools in several areas, including healthcare, for example, iOS applications.

We started this research project by conducting an extensive study at available gene and disease annotation repositories and resources for research and clinical purposes.^1^ We enhanced the scope of our research project with the implementation of a new gene‐disease database (PAS‐Gen).^136^ The goal was to collect all distinct, authentic, and actionable genes‐based information and related diseases from maximum possible available sources. In this paper, we have presented further extended research to our research project, with the inclusion of over a million somatic mutations, over 100 thousand germline mutations, and available clinical relevant drug and disease codes, and their variable associations. In the future, we are looking forward to extending the scope of our database and application by adding more useful data science and visualization features. We will implement multifunctional modules based on machine learning algorithms, which will help clinical and genomics data integration and facilitate natural language processing‐based search. Currently, we are focused on Homo sapiens and in the future, we will be extending our research and development, including available genomes of other species, especially *Mus musculus*, *Drosophila melanogaster*, and microbes. Furthermore, we are looking forward to expanding our research project with the development of new project web page and online tools.

## 5 CONCLUSIONS

This is the era of Big Data, where human‐related biological databases continue to grow not only in count but also in volume, posing unprecedented challenges in data storage, processing, exchange, and curation. We developed a cutting‐edge gene‐SNP‐disease‐drug database accessible through a smart phone application, integrating information about classified diseases and related genes, germline and somatic mutations, and drugs. The study focused on developing a tool that might help others (mainly researchers, medical practitioners, and pharmacists) in having a broad view of genetic variants that may be implicated in the likelihood of developing certain diseases.^1^ We have developed a platform that can provide new understandings into the information about genetic basis of human complex diseases and contribute to assimilating genomic with phenotypic data for the availability of gene‐based designer drugs, precise targeting of molecular fingerprints for tumor, appropriate drug therapy, predicting individual susceptibility to disease, diagnosis and treatment of rare illnesses are all a few of the many transformations expected in the decade to come.

## DECLARATIONS

### COMPETING INTERESTS

The authors declare that they have no competing interests.

## Supporting information

Additional file 1: “PAS Guide and Workflow Design.”

Additional file 2: “Supplementary Figure 3.”

Additional file 3: “Supplementary Figure 4.”

Additional file 4: “Supplementary Figure 5.”

Additional file 5: “Supplementary Figure 6.”
