# Supplementary material for: Human gene and disease associations for clinical‐genomics and precision medicine research
Source: Clin Transl Med. 2020 May 3;10(1):297–318. doi: 10.1002/ctm2.28 (PMC7240856; doi:10.1002/ctm2.28)
Supplement: Supplementary file 1 — Additional file 1: “PAS Guide and Workflow Design.” [file CTM2-10-297-s001.pdf]

Supplementary Material: Human Gene and Disease Associations for Clinical-Genomics and Precision Medicine Research

PAS Guide and Workflow Design

Zeeshan Ahmed[1, 2, *], Saman Zeeshan[3], Dinesh Mendhe[1] and XinQi Dong[1, 2]

[1]Institute for Health, Health Care Policy and Aging Research, Rutgers, The State University of New Jersey, 112 Paterson Street, New Brunswick, NJ, USA.

[2]Department of Medicine, Rutgers Robert Wood Johnson Medical School, Rutgers Biomedical and Health Sciences, 125 Paterson Street, New Brunswick, NJ, USA.

[3]Rutgers Cancer Institute of New Jersey, Rutgers, The State University of New Jersey, 112 Paterson Street, New Brunswick, NJ, USA.

[*]Corresponding author. Institute for Health, Health Care Policy and Aging Research, Rutgers, The State University of New Jersey, 112 Paterson Street, New Brunswick, NJ, USA.

Email: zahmed@ifh.rutgers.edu

PAS

PAS is an iOS app suite to assist clinical and life science's researchers by providing larger exposure to the authentic genes and their association to classified diseases with greater visibility and easy one tap browsing, saving time in scanning through genes and developing gene-disease lists for a research study. PAS is an app developed on iPhone Operating System (iOS) platform (version 12.1). PAS graphical user interfaces are developed using Swift multi-paradigm programming language and XCODE (version 10) integrated development environment for macOS. The database is modelled and hosted within MySQL database management system. It has dynamic, web based pages to facilitate data migration between app and database are developed PHP scripting language (S. Figure 1).

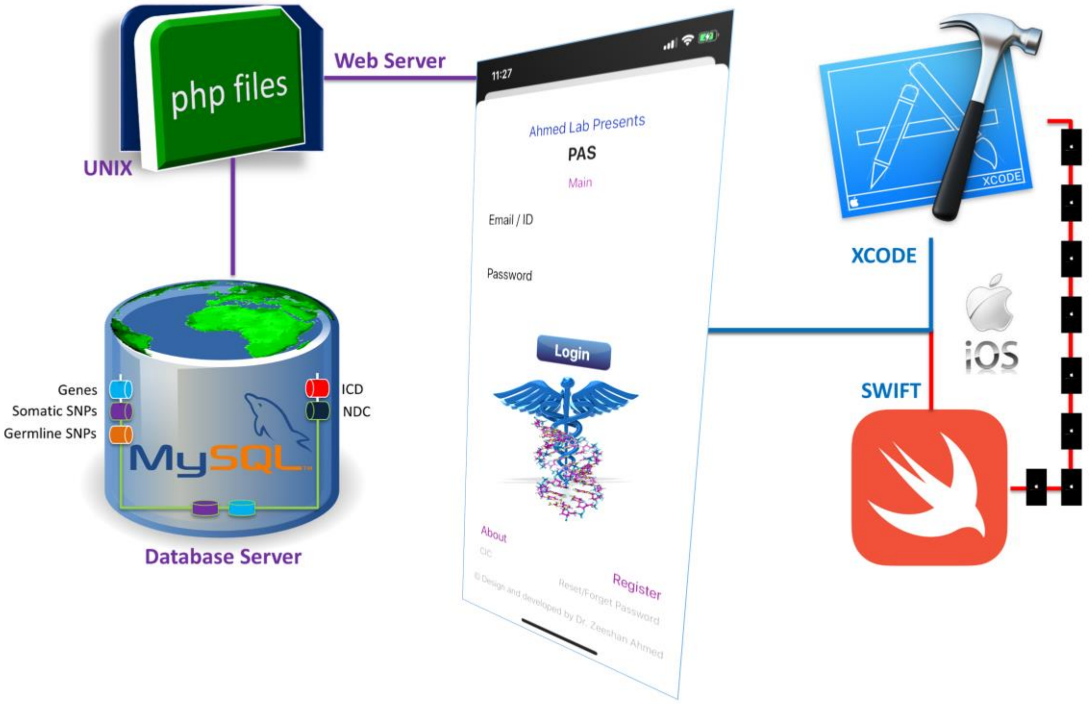

S. Figure 1. PAS components design, development, and data flow.

PAS is based on product line architecture (PLA), designed following the Butterfly model [1, 2, 3], with all major modules capable of performing individual key roles and can integrate with each other. One of the most difficult and complex tasks of implementing an Apple mobile app connecting via PHP programmed modules to an external web-based MySQL server for data exchange, is the integration of all modules developed using different programming languages and processed through different compilers/interpreters that sometimes cause non-syntax logical errors, which are hard to debug. PAS was mainly tested using XCODE-provided simulator and Apple iPhone 8, X and iPad mobile devices with the most recent iOS version (12.1). It is reviewed and approved by the Apple, and freely available to download at the App Store.

PAS: Main

This is the first launched, main (S. Figure 2) user interface of the PAS app, leading to five other sub-interfaces for taking different inputs from the user, allowing user to perform different operations and sharing important information.

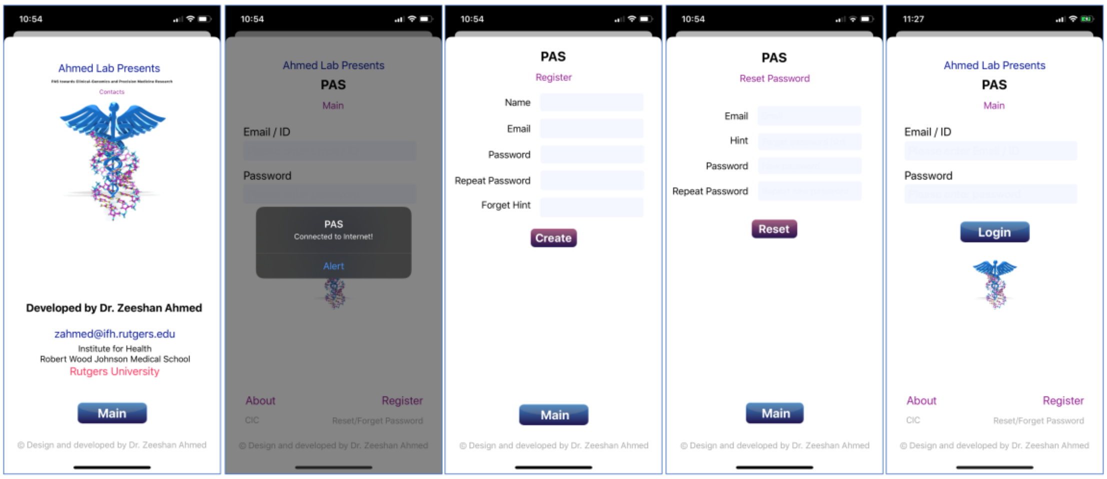

A: About B: Check internet connection C: Register D: Reset Password E: Login

S. Figure 2. PAS: About, Main, Register, Reset Password, and Main.

Main interface provides following six features:

1. Main allows user to navigate to the "About" interface (S. Figure 2A) by pressing button "About", which provides information about the app and author's contacts.
2. PAS requires internet as a mandatory requirement, Main allows user to check if the iPhone device is successfully connected to the available internet service (S. Figure 2B) by pressing button "CiC".
3. Main allows user to navigate to user registration interface (S. Figure 2C) by pressing "Register" button.
4. Main allows user to navigate to change password interface (S. Figure 2D) by pressing "Reset Password" button.
5. Main allows user to enter user name and password, and press "Login" button. Having valid user credentials, it automatically navigates to the Menu interface (S. Figure 2E).

Register (S. Figure 2C) interface allows user to create new login for using PAS. It requires user to enter seven following values:

1. Full name of the user.
2. Email of the user, which will also be the user's ID for PAS.
3. Password
4. Repeat password to make sure, user's entered is the user's expected password.
5. Forget hint, to use when password is forgotten and need to reset password.

After entering requested information, user is required to press "Create" button to register, and press "Main" button to navigate back to the Main interface.Reset Password

(S. Figure 2D) interface allows user to reset the password. It requires user to enter following four data entries:

1. Email address, entered by the user to register.
2. Forget password hint, entered at the time user registered.
3. New password.
4. Repeat password to make sure, user's entered is the user's expected password.

After entering requested information, user is required to press "Reset" button to set new password, and press "Main" button to navigate back to the Main interface.

Menu

Menu interface allows user to navigate to Clinics, Genomics and Clinical Genomics interfaces (S. Figure 5).

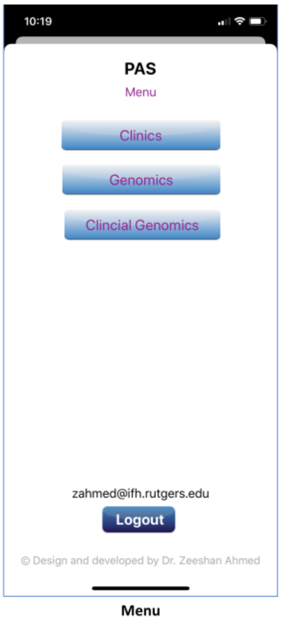

S. Figure 3. PAS Menu: Clinics, Genomics and Clinical Genomics

PAS: Clinics

Clinics interface (S. Figure 4A) provides four options to navigate to International Classifications of Diseases (ICD), ICD-10, ICD-9 and National Drug Codes (NDC). International Classifications of Diseases (ICD) (S. Figure 4B) provides white input text field and allows user to enter complete or partial ICD code, Disease name and related description and search by pressing "Search" button. The results are presented in the gray text box, and the total number of relevant records found are provided in the text field below. Moreover, ICD provides "Menu" button to navigate back to the Menu interface. ICD-10 (S. Figure 4C) provides a white input text field and allows user to enter complete or partial ICD-10 code, Disease name and related description and search by pressing "Search" button. The results are presented in gray text box, and the total number of relevant records found are given in below text field. Moreover, ICD-10 provides "Menu" button to navigate back to the Menu interface. ICD-9 (S. Figure 4D) provides a white text input field and allows user to enter complete or partial ICD-9 code,

Disease name and related description and search by pressing “Search” button. The results are presented in gray text box, and total number of relevant records found are given in below text field. Moreover, ICD-9 provides “Menu” button to navigate back to the Menu interface.

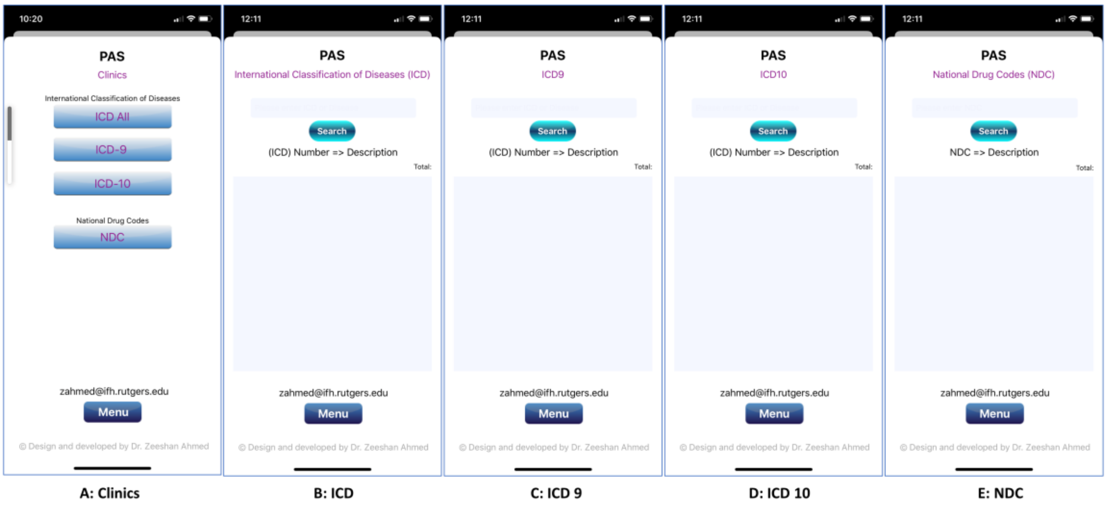

S. Figure 4. PAS: Clinics, ICD, ICD9, ICD10, NDC

National Drug Codes (NDC) (S. Figure 4E) provides a white text input field and allows user to enter complete or partial NDC, Proprietary and related description, and search by pressing “Search” button. The results are presented in pink text box, and total number of relevant records found are given in below text field. Moreover, NDC provides “Menu” button to navigate back to the Menu interface. The NDC interface enables users to search for NDC and related details, which includes: Product ID, Product NDC, Product Type Name, Proprietary Name, Non-Proprietary Name, Dosage Form Name, Route Name, Marketing Category Name, Application Number, Labeler Name, Substance Name, Active Numerator Strength, Active Ingred Unit, Pharm Classes, and Listing Records Certified Through. The PAS graphical user interface enables users to search by complete or partial word matching.

PAS: Genomics

Genomics interface (S. Figure 5A) provides three options to navigate to Gene, SNPs and Somatic SNPs. Genes (S. Figure 5B) provides white input text field and allows user to enter complete or partial gene name or ensemble id and search by pressing “Search” button. The results are presented in the green text box, and the total number of relevant records found are provided in the text field below. Moreover, Genes provides “Menu” button to navigate back to the Menu interface. The Genes’ interface enables users to search for Genes and related details, which includes: Gene Name, Ensemble ID, Type and Chromosome. The PAS graphical user interface enables users to search by complete or partial word matching.
User needs to navigate from Menu to Genomics, and then to SNPs interface (S. Figure 5C). SNPs provides white input text field and allows user to enter complete or partial

SNP name or ensemble id and search by pressing “Search” button. The results are presented in the blue text box, and the total number of relevant records found are provided in the text field below. Moreover, SNPs provides “Menu” button to navigate back to the Menu interface. The SNPs’ interface enables users to search for SNPs and related details, which includes: SNP, Reported Gene, Mapped Gene, Chromosome, Chromosome Position, Region, Context, Platform Affymetrix and PUBMED ID. The PAS graphical user interface enables users to search by complete or partial word matching.

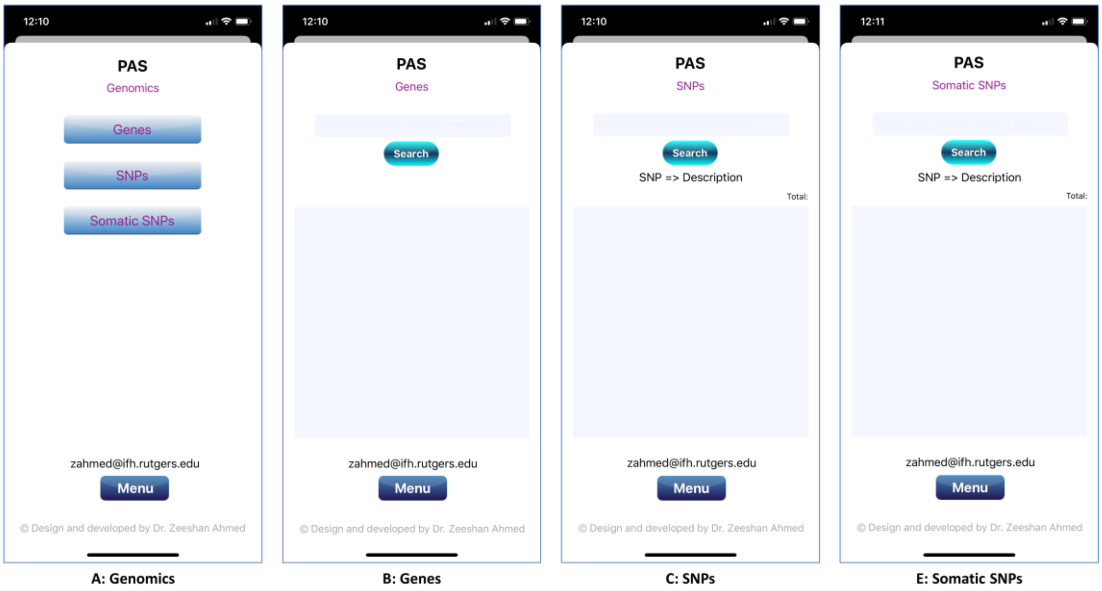

S. Figure 5. PAS: Genomics, Genes, SNPs, Somatic SNPs.

User needs to navigate from Menu to Genomics, and then to Somatic SNPs interface (S. Figure 5D). Somatic SNPs provides white input text field and allows user to enter complete or partial SNP name or ensemble id and search by pressing “Search” button. The results are presented in the blue text box, and the total number of relevant records found are provided in the text field below. Moreover, Somatic SNPs provides “Menu” button to navigate back to the Menu interface. The Somatic SNPs’ interface enables users to search for Genes and SNPs, and related details, which includes: Reported gene, Accession number, ID tumor, Gene CDS length, Sample name, ID sample, Primary site, Site subtype, Mutation AA, Mutation description, Mutation CDS, GRCH, Mutation genome position, FATHMM prediction, FATHMM score, Mutation somatic status, Sample type, Tumor origin, ID study, Age and PUBMED ID. The PAS graphical user interface enables users to search by complete or partial word matching.

PAS: Clinical Genomics

Clinical Genomics interface (S. Figure 6A) provides three options to navigate to Gene, SNPs and Somatic SNPs. User needs to navigate from Menu to Clinical Genomics, and then to Gene to Disease interface (S. Figure 6B). Gene to Disease provides a white input text field and allows user to enter complete or partial gene and disease name and search by pressing “Search” button. The results are presented in red text box, and the total number of relevant records found are given in below text field. Moreover, Gene to Disease provides “Menu” button to navigate back to the Menu interface. The Genes to

Disease' interface enables users to search for Genes, Disease and related details, which includes: Gene Name, Ensemble ID, Type, Disease and Chromosome. The PAS graphical user interface enables users to search by complete or partial word matching. The gene-disease querying ability offered by PAS provides the user with an important knowledge discovery tool, just a click away from any location.

User needs to navigate from Menu to Clinical Genomics, and then to SNP to Disease interface (S. Figure 6C). SNP to Disease provides a white input text field and allows user to enter complete or partial SNP and disease name and search by pressing "Search" button. The results are presented in blue text box, and the total number of relevant records found are given in below text field. Moreover, SNP to Disease provides "Menu" button to navigate back to the Menu interface. The SNPs to Disease' interface enables users to search for SNPs, Disease and related details, which includes: SNP, Reported Gene, Mapped Gene, Chromosome, Context, Disease and Study. The PAS graphical user interface enables users to search by complete or partial word matching. The SNP-disease querying ability offered by PAS provides the user with an important knowledge discovery tool, just a click away from any location.

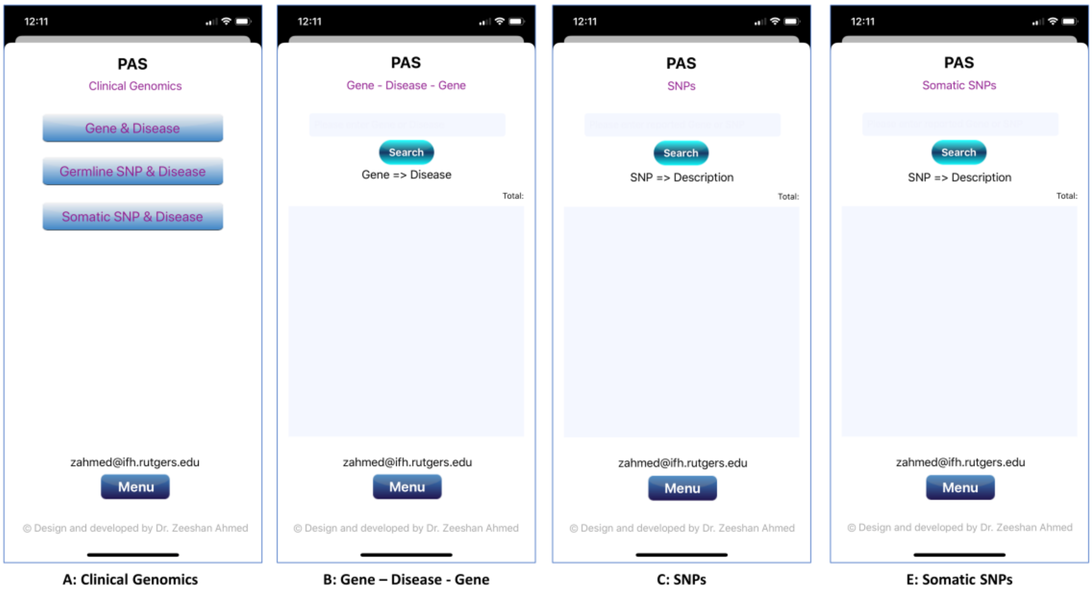

S. Figure 6. PAS Clinical Genomics. Gene-Disease-Gene, SNPs, Somatic SNPs.

User needs to navigate from Menu to Clinical Genomics, and then to Somatic SNP & Disease interface (S. Figure 6D). Somatic SNP & Disease provides a white input text field and allows user to enter complete or partial SNP and disease name and search by pressing "Search" button. The results are presented in blue text box, and the total number of relevant records found are given in below text field. Moreover, Somatic SNP & Disease provides "Menu" button to navigate back to the Menu interface. The Somatic SNPs & Disease' interface enables users to search for Gene, SNPs, Disease and related details, which includes: Reported gene, Primary site, Primary histology, Histology subtype, Mutation ID, Mutation description, Mutation genome position, Accession number. The PAS graphical user interface enables users to search by complete or partial word matching. The Somatic SNP & Disease querying ability offered by PAS provides the user with an important knowledge discovery tool, just a click away from any location.

References

1. Ahmed Z, Zeeshan S, Dandekar T. Developing sustainable software solutions for bioinformatics by the “Butterfly” paradigm. F1000Research 2014; 7:54-66.
2. Ahmed Z, Zeeshan S. Cultivating Software Solutions Development in the Scientific Academia. Recent Patents on Computer Science 2014; 7:54-66.
3. Ahmed Z. Designing Flexible GUI to Increase the Acceptance Rate of Product Data Management Systems in Industry. International Journal of Computer Science & Emerging Technologies 2011; 2:100-109.
