# Supplementary material for: Human gene and disease associations for clinical‐genomics and precision medicine research
Source: Clin Transl Med. 2020 May 3;10(1):297–318. doi: 10.1002/ctm2.28 (PMC7240856; doi:10.1002/ctm2.28)
Supplement: Supplementary file 2 — Additional file 2: “Supplementary Figure 3.” [file CTM2-10-297-s002.pdf]

10:02

PAS

Gene - Disease - Gene

chlamydia

Search

Gene => Disease

Total: 22

Gene: TNF
-Ensembl ID: ENSG00000232810
-Type: Protein Coding
-Disease: chlamydia
-Chromosome: chr6

Gene: IL6
-Ensembl ID: ENSG00000136244
-Type: Protein Coding
-Disease: chlamydia
-Chromosome: chr7

Gene: IL1B
-Ensembl ID: ENSG00000125538
-Type: Protein Coding
-Disease: chlamydia

zahmed@ifh.rutgers.edu

Menu

© Design and developed by Dr. Zeeshan Ahmed

10:03

PAS

Gene - Disease - Gene

influenza

Search

Gene => Disease

Total: 86

Gene: TNF
-Ensembl ID: ENSG00000232810
-Type: Protein Coding
-Disease: haemophilus_influenzae
-Chromosome: chr6

Gene: TNF
-Ensembl ID: ENSG00000232810
-Type: Protein Coding
-Disease: avian_influenza
-Chromosome: chr6

Gene: TNF
-Ensembl ID: ENSG00000232810
-Type: Protein Coding
-Disease: swine_influenza

zahmed@ifh.rutgers.edu

Menu

© Design and developed by Dr. Zeeshan Ahmed

10:03

PAS

Gene - Disease - Gene

staph

Search

Gene => Disease

Total: 31

Gene: TNF
-Ensembl ID: ENSG00000232810
-Type: Protein Coding
-Disease: staphylococcal_toxic_shock_syndrome
-Chromosome: chr6

Gene: IL1B
-Ensembl ID: ENSG00000125538
-Type: Protein Coding
-Disease: staphylococcal_toxic_shock_syndrome
-Chromosome: chr2

Gene: IFNG
-Ensembl ID: ENSG00000111537
-Type: Protein Coding
-Disease: staphylococcal_toxic_shock_syndrome

zahmed@ifh.rutgers.edu

Menu

© Design and developed by Dr. Zeeshan Ahmed

10:04

PAS

Gene - Disease - Gene

herpes

Search

Gene => Disease

Total: 103

Gene: IL6
-Ensembl ID: ENSG00000136244
-Type: Protein Coding
-Disease: human_herpesvirus_8
-Chromosome: chr7

Gene: APOE
-Ensembl ID: ENSG00000130203
-Type: Protein Coding
-Disease: herpes_simplex_encephalitis
-Chromosome: chr19

Gene: APOE
-Ensembl ID: ENSG00000130203
-Type: Protein Coding
-Disease: genital_herpes

zahmed@ifh.rutgers.edu

Menu

© Design and developed by Dr. Zeeshan Ahmed

10:05

PAS

Gene - Disease - Gene

shigellosis

Search

Gene => Disease

Total: 16

Gene: IL10
-Ensembl ID: ENSG00000136634
-Type: Protein Coding
-Disease: shigellosis
-Chromosome: chr1

Gene: CXCL8
-Ensembl ID: ENSG00000169429
-Type: Protein Coding
-Disease: shigellosis
-Chromosome: chr4

Gene: SRC
-Ensembl ID: ENSG00000197122
-Type: Protein Coding
-Disease: shigellosis

zahmed@ifh.rutgers.edu

Menu

© Design and developed by Dr. Zeeshan Ahmed

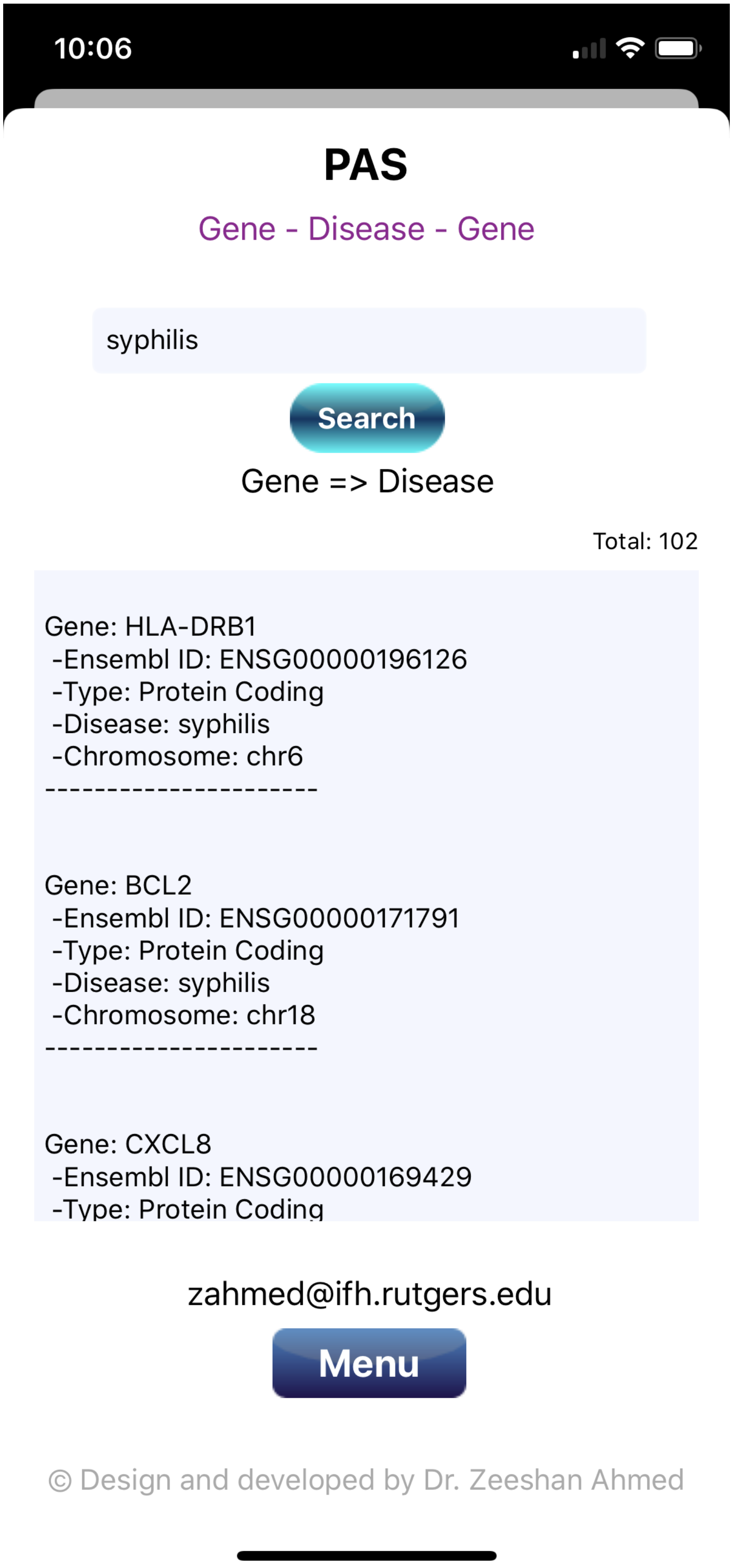
10:06
PAS
Gene - Disease - Gene
syphilis
Search
Gene => Disease
Total: 102
Gene: HLA-DRB1
-Ensembl ID: ENSG00000196126
-Type: Protein Coding
-Disease: syphilis
-Chromosome: chr6

Gene: BCL2
-Ensembl ID: ENSG00000171791
-Type: Protein Coding
-Disease: syphilis
-Chromosome: chr18

Gene: CXCL8
-Ensembl ID: ENSG00000169429
-Type: Protein Coding
zahmed@ifh.rutgers.edu
Menu
© Design and developed by Dr. Zeeshan Ahmed

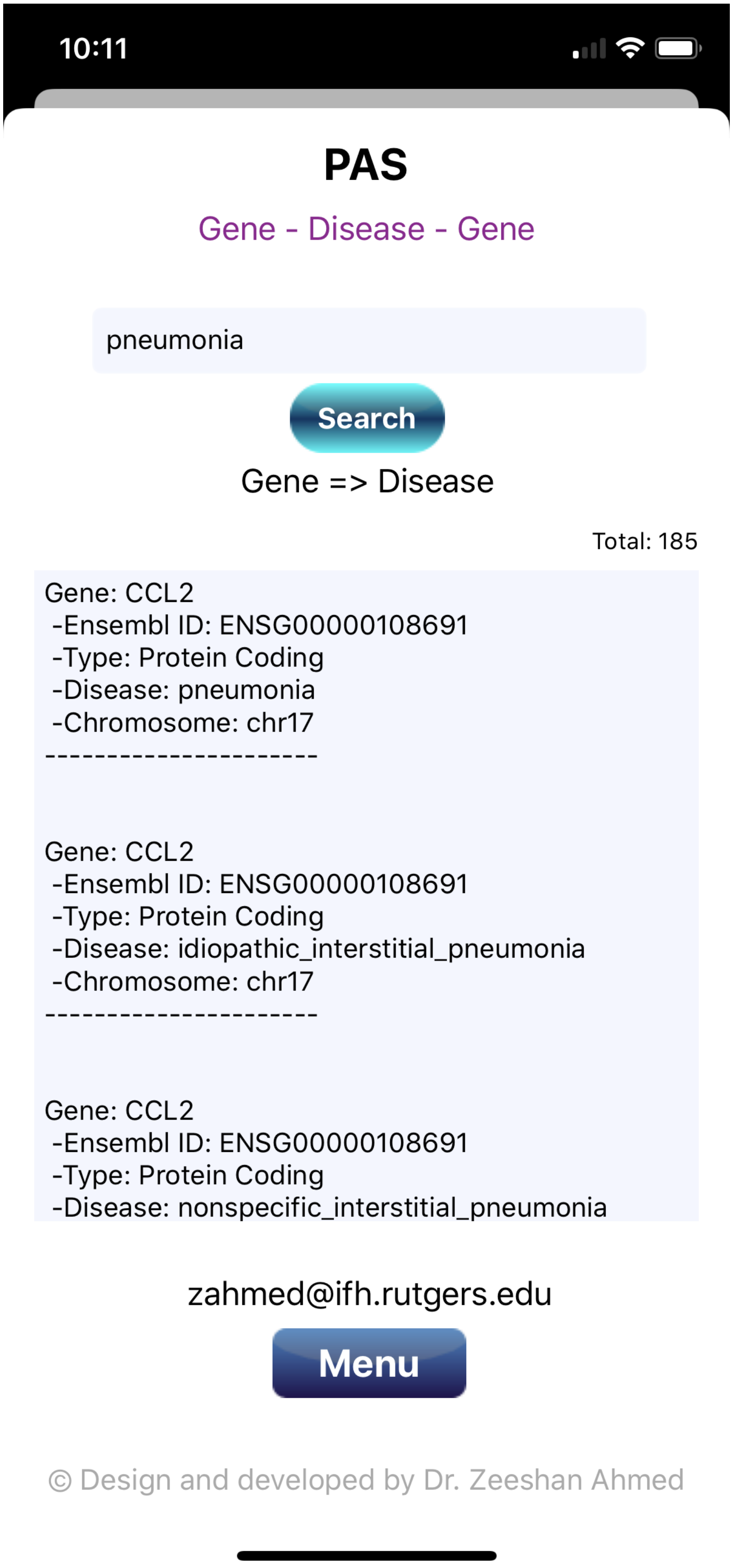
10:11
PAS
Gene - Disease - Gene
pneumonia
Search
Gene => Disease
Total: 185
Gene: CCL2
-Ensembl ID: ENSG00000108691
-Type: Protein Coding
-Disease: pneumonia
-Chromosome: chr17

Gene: CCL2
-Ensembl ID: ENSG00000108691
-Type: Protein Coding
-Disease: idiopathic_interstitial_pneumonia
-Chromosome: chr17

Gene: CCL2
-Ensembl ID: ENSG00000108691
-Type: Protein Coding
-Disease: nonspecific_interstitial_pneumonia
zahmed@ifh.rutgers.edu
Menu
© Design and developed by Dr. Zeeshan Ahmed

10:11

PAS

Gene - Disease - Gene

hepatitis

Search

Gene => Disease

Total: 325

Gene: TNF
-Ensembl ID: ENSG00000232810
-Type: Protein Coding
-Disease: hepatitis
-Chromosome: chr6

Gene: TNF
-Ensembl ID: ENSG00000232810
-Type: Protein Coding
-Disease: hepatitis_a
-Chromosome: chr6

Gene: TNF
-Ensembl ID: ENSG00000232810
-Type: Protein Coding
-Disease: hepatitis_c

zahmed@ifh.rutgers.edu

Menu

© Design and developed by Dr. Zeeshan Ahmed

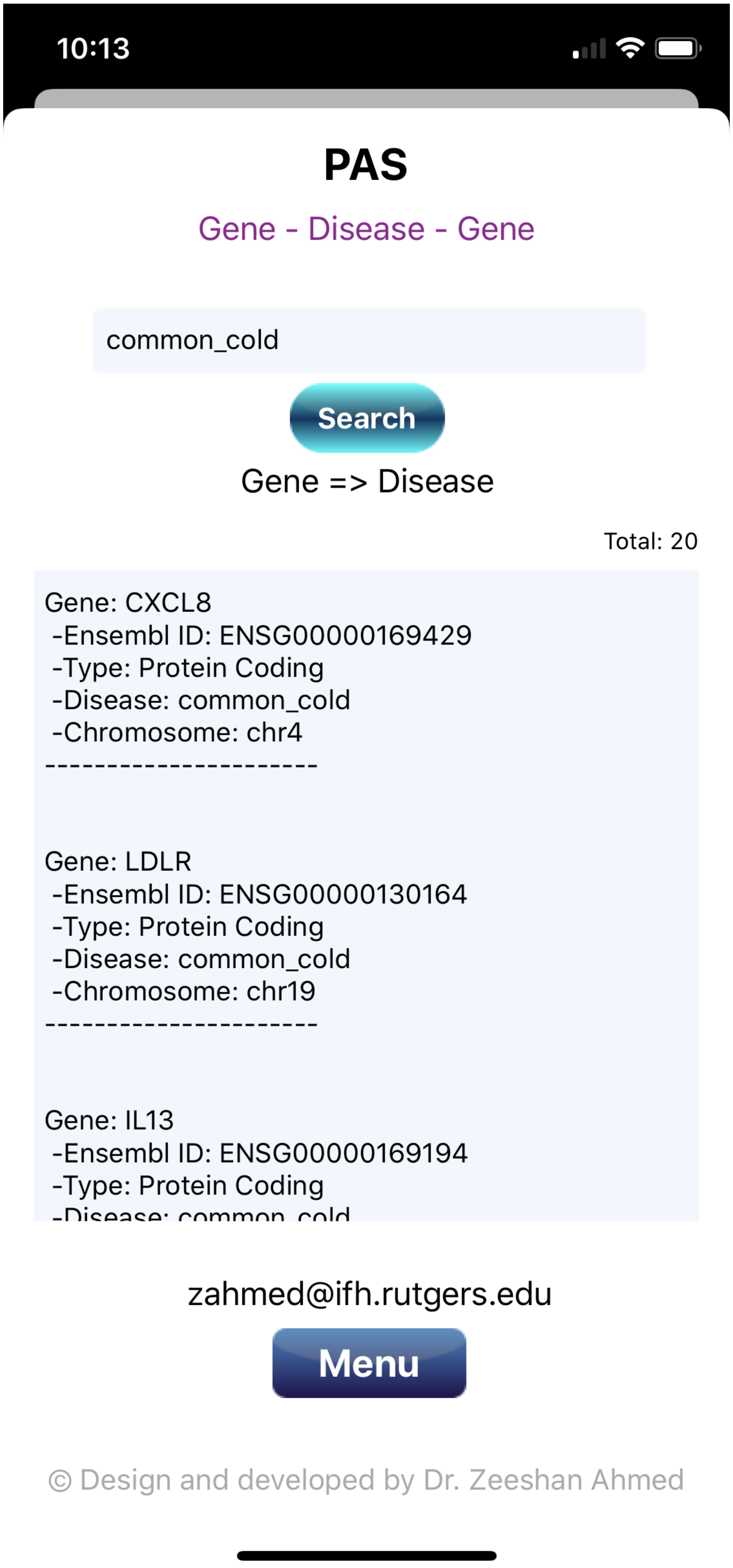
10:13
PAS
Gene - Disease - Gene
common_cold
Search
Gene => Disease
Total: 20
Gene: CXCL8
-Ensembl ID: ENSG00000169429
-Type: Protein Coding
-Disease: common_cold
-Chromosome: chr4

Gene: LDLR
-Ensembl ID: ENSG00000130164
-Type: Protein Coding
-Disease: common_cold
-Chromosome: chr19

Gene: IL13
-Ensembl ID: ENSG00000169194
-Type: Protein Coding
zahmed@ifh.rutgers.edu
Menu
© Design and developed by Dr. Zeeshan Ahmed

10:17

PAS

Gene - Disease - Gene

salmonellosis

Search

Gene => Disease

Total: 17

-Disease: salmonellosis
-Chromosome: chr1

Gene: TLR4
-Ensembl ID: ENSG00000136869
-Type: Protein Coding
-Disease: salmonellosis
-Chromosome: chr9

Gene: STAT1
-Ensembl ID: ENSG00000115415
-Type: Protein Coding
-Disease: salmonellosis
-Chromosome: chr2
----

zahmed@ifh.rutgers.edu

Menu

© Design and developed by Dr. Zeeshan Ahmed
