# Supplementary material for: Human gene and disease associations for clinical‐genomics and precision medicine research
Source: Clin Transl Med. 2020 May 3;10(1):297–318. doi: 10.1002/ctm2.28 (PMC7240856; doi:10.1002/ctm2.28)
Supplement: Supplementary file 3 — Additional file 3: “Supplementary Figure 4.” [file CTM2-10-297-s003.pdf]

10:41

PAS

SNPs

diabetes

Search

SNP => Description

Total: 2174

Reported Gene: KCNQ1
-SNP: rs163182
-Chromosome: 11
-Context: intron_variant
-Disease: Type 2 diabetes
-Study: A genome-wide association study confirms previously reported loci for type 2 diabetes in Han Chinese.

Reported Gene: C2CD4B, C2CD4A
-SNP: rs1436953
-Chromosome: 15
-Context: intergenic_variant
-Disease: Type 2 diabetes
-Study: A genome-wide association study confirms previously reported loci for type 2 diabetes in Han Chinese.
----

zahmed@ifh.rutgers.edu

Menu

© Design and developed by Dr. Zeeshan Ahmed

10:41

PAS

SNPs

immune

Search

SNP => Description

Total: 2583

Reported Gene: HLA-B
-SNP: rs2524054
-Chromosome: 6
-Context: intron_variant
-Disease: CD4:CD8 lymphocyte ratio
-Study: Quantitative trait loci for CD4:CD8 lymphocyte ratio are associated with risk of type 1 diabetes and HIV-1 immune control.

Reported Gene: HLA-B
-SNP: rs2524054
-Chromosome: 6
-Context: intron_variant
-Disease: CD4:CD8 lymphocyte ratio
-Study: Quantitative trait loci for CD4:CD8 lymphocyte ratio are associated with risk of type 1 diabetes and HIV-1 immune control.
----

zahmed@ifh.rutgers.edu

Menu

© Design and developed by Dr. Zeeshan Ahmed

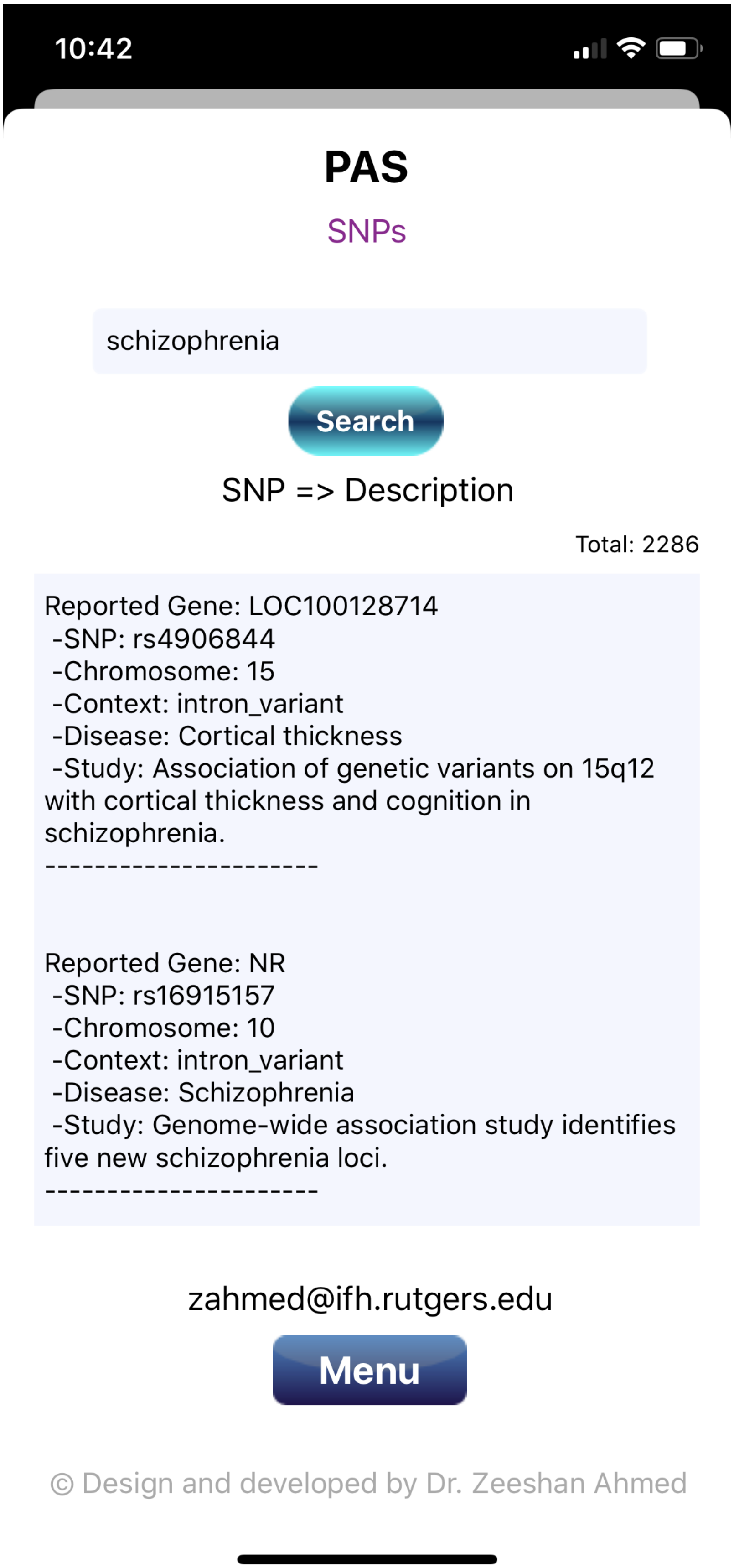
10:42
PAS
SNPs
schizophrenia
Search
SNP => Description
Total: 2286
Reported Gene: LOC100128714
-SNP: rs4906844
-Chromosome: 15
-Context: intron_variant
-Disease: Cortical thickness
-Study: Association of genetic variants on 15q12 with cortical thickness and cognition in schizophrenia.

Reported Gene: NR
-SNP: rs16915157
-Chromosome: 10
-Context: intron_variant
-Disease: Schizophrenia
-Study: Genome-wide association study identifies five new schizophrenia loci.
----
zahmed@ifh.rutgers.edu
Menu
© Design and developed by Dr. Zeeshan Ahmed

10:42

PAS

SNPs

hla-drb1

Search

SNP => Description

Total: 216

Reported Gene: HLA-DRB1, HLA-DQA1
 -SNP: rs3129763
 -Chromosome: 6
 -Context: regulatory_region_variant
 -Disease: Systemic sclerosis
 -Study: Identification of novel genetic markers associated with clinical phenotypes of systemic sclerosis through a genome-wide association strategy.

Reported Gene: HLA-DRB1
 -SNP: rs9271366
 -Chromosome: 6
 -Context: intergenic_variant
 -Disease: Immunoglobulin A
 -Study: Association of IFIH1 and other autoimmunity risk alleles with selective IgA deficiency

zahmed@ifh.rutgers.edu

Menu

© Design and developed by Dr. Zeeshan Ahmed

10:43

PAS

SNPs

tnfrsf1a

Search

SNP => Description

Total: 35

Reported Gene: HLA-DRB1
 -SNP: rs3135388
 -Chromosome: 6
 -Context: intergenic_variant
 -Disease: Multiple sclerosis
 -Study: Meta-analysis of genome scans and replication identify CD6, IRF8 and TNFRSF1A as new multiple sclerosis susceptibility loci.

Reported Gene: HLA-B
 -SNP: rs2523393
 -Chromosome: 6
 -Context: non_coding_transcript_exon_variant
 -Disease: Multiple sclerosis
 -Study: Meta-analysis of genome scans and replication identify CD6, IRF8 and TNFRSF1A as new multiple sclerosis susceptibility loci.
----

zahmed@ifh.rutgers.edu

Menu

© Design and developed by Dr. Zeeshan Ahmed

10:43

PAS

SNPs

ptpn22

Search

SNP => Description

Total: 44

Reported Gene: PTPN22
-SNP: rs2476601
-Chromosome: 1
-Context: missense_variant
-Disease: Crohns disease
-Study: Genome-wide association defines more than 30 distinct susceptibility loci for Crohns disease.

Reported Gene: PTPN22
-SNP: rs2476601
-Chromosome: 1
-Context: missense_variant
-Disease: Type 1 diabetes
-Study: A genome-wide association study identifies KIAA0350 as a type 1 diabetes gene.
----

zahmed@ifh.rutgers.edu

Menu

© Design and developed by Dr. Zeeshan Ahmed

10:44

PAS

SNPs

hnf1

Search

SNP => Description

Total: 142

Reported Gene: HNF1A
 -SNP: rs2393791
 -Chromosome: 12
 -Context: intron_variant
 -Disease: Gamma glutamyl transpeptidase
 -Study: Large-scale genome-wide association studies in East Asians identify new genetic loci influencing metabolic traits.

Reported Gene: C12orf27, HNF1A
 -SNP: rs7310409
 -Chromosome: 12
 -Context: intron_variant
 -Disease: Liver enzyme levels (gamma-glutamyl transferase)
 -Study: Genome-wide association study identifies loci influencing concentrations of liver enzymes in plasma

zahmed@ifh.rutgers.edu

Menu

© Design and developed by Dr. Zeeshan Ahmed

10:44

PAS

SNPs

hnf4a

Search

SNP => Description

Total: 46

Reported Gene: HNF4A
-SNP: rs4812829
-Chromosome: 20
-Context: intron_variant
-Disease: Type 2 diabetes
-Study: Genome-wide association study in individuals of South Asian ancestry identifies six new type 2 diabetes susceptibility loci.

Reported Gene: HNF4A
-SNP: rs1800961
-Chromosome: 20
-Context: missense_variant
-Disease: HDL cholesterol
-Study: Biological, clinical and population relevance of 95 loci for blood lipids.
----

zahmed@ifh.rutgers.edu

Menu

© Design and developed by Dr. Zeeshan Ahmed

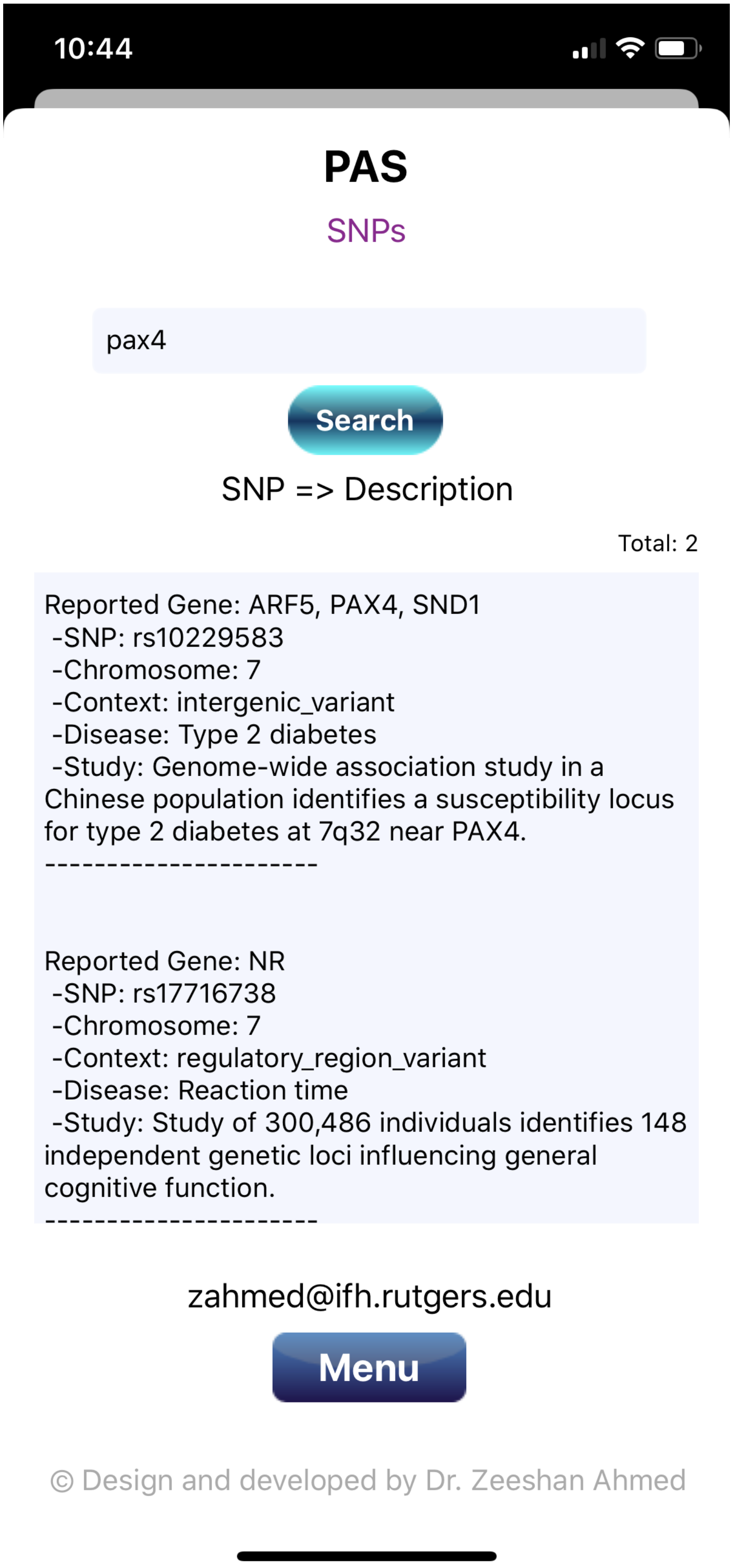
10:44
PAS
SNPs
pax4
Search
SNP => Description
Total: 2
Reported Gene: ARF5, PAX4, SND1
-SNP: rs10229583
-Chromosome: 7
-Context: intergenic_variant
-Disease: Type 2 diabetes
-Study: Genome-wide association study in a Chinese population identifies a susceptibility locus for type 2 diabetes at 7q32 near PAX4.

Reported Gene: NR
-SNP: rs17716738
-Chromosome: 7
-Context: regulatory_region_variant
-Disease: Reaction time
-Study: Study of 300,486 individuals identifies 148 independent genetic loci influencing general cognitive function.
----
zahmed@ifh.rutgers.edu
Menu
© Design and developed by Dr. Zeeshan Ahmed

10:44

PAS

SNPs

drd2

Search

SNP => Description

Total: 10

Reported Gene: DRD2
-SNP: rs11214606
-Chromosome: 11
-Context: intron_variant
-Disease: Response to antipsychotic treatment in schizophrenia (working memory)
-Study: Genome-wide pharmacogenomic study of neurocognition as an indicator of antipsychotic treatment response in schizophrenia.

Reported Gene: DRD2
-SNP: rs1079596
-Chromosome: 11
-Context: intron_variant
-Disease: Select biomarker traits
-Study: Genome-wide association with select biomarker traits in the Framingham Heart Study.
----

zahmed@ifh.rutgers.edu

Menu

© Design and developed by Dr. Zeeshan Ahmed

10:45

PAS

SNPs

cacnb2

Search

SNP => Description

Total: 45

Reported Gene: CACNB2
-SNP: rs12258967
-Chromosome: 10
-Context: intron_variant
-Disease: Blood pressure
-Study: Genome-wide association study identifies six new loci influencing pulse pressure and mean arterial pressure.

Reported Gene: CACNB2
-SNP: rs4373814
-Chromosome: 10
-Context: intergenic_variant
-Disease: Systolic blood pressure
-Study: Genetic variants in novel pathways influence blood pressure and cardiovascular disease risk.
----

zahmed@ifh.rutgers.edu

Menu

© Design and developed by Dr. Zeeshan Ahmed

10:45

PAS

SNPs

grm3

Search

SNP => Description

Total: 16

Reported Gene: NR
-SNP: rs2189812
-Chromosome: 7
-Context: intron_variant
-Disease: IgG glycosylation
-Study: Loci associated with N-glycosylation of human immunoglobulin G show pleiotropy with autoimmune diseases and haematological cancers.

Reported Gene: NR
-SNP: rs10952890
-Chromosome: 7
-Context: intron_variant
-Disease: Gestational age at birth in labor-initiated deliveries (child effect)
-Study: Literature-Informed Analysis of a Genome-Wide Association Study of Gestational Age in Norwegian Women and Children Suggests

zahmed@ifh.rutgers.edu

Menu

© Design and developed by Dr. Zeeshan Ahmed
