# Supplementary material for: Human gene and disease associations for clinical‐genomics and precision medicine research
Source: Clin Transl Med. 2020 May 3;10(1):297–318. doi: 10.1002/ctm2.28 (PMC7240856; doi:10.1002/ctm2.28)
Supplement: Supplementary file 4 — Additional file 4: “Supplementary Figure 5.” [file CTM2-10-297-s004.pdf]

10:51

PAS

Somatic SNPs

esr1

Search

SNP => Description

Total: 216

Reported Gene: ESR1_ENST00000544394
-Accession Number: ENST00000544394
-ID tumour: 2577643
-let_Gene CDS length: 933
-HGNC ID: NA
-let_Sample_name: P-0002562-T01-IM3
-ID sample: 2718822
-Primary site: breast
-Mutation AA: p.E207Q
-Mutation Description: Substitution - Missense
-Mutation CDS: c.619G>C
-GRCh: 38
-Mutation genome position: 6:152011697-152011697
-FATHMM prediction: PATHOGENIC
-FATHMM score: 0.99994
-Mutation somatic status: Confirmed somatic variant
-Sample Type: fixed - NOS
-Tumour origin: metastasis

zahmed@ifh.rutgers.edu

Menu

© Design and developed by Dr. Zeeshan Ahmed

10:52

PAS

Somatic SNPs

akt1

Search

SNP => Description

Total: 178

Reported Gene: AKT1
-Accession Number: ENST00000349310
-ID tumour: 2527787
-let_Gene CDS length: 1443
-HGNC ID: 391
-let_Sample_name: BC80-tumour
-ID sample: 2668208
-Primary site: breast
-Mutation AA: p.E17K
-Mutation Description: Substitution - Missense
-Mutation CDS: c.49G>A
-GRCh: 38
-Mutation genome position: 14:104780214-104780214
-FATHMM prediction: PATHOGENIC
-FATHMM score: 0.99513
-Mutation somatic status: Confirmed somatic variant
-Sample Type: surgery fresh/frozen
-Tumour origin: metastasis

zahmed@ifh.rutgers.edu

Menu

© Design and developed by Dr. Zeeshan Ahmed

10:52

PAS

Somatic SNPs

erbb2

Search

SNP => Description

Total: 600

Reported Gene: ERBB2IP_ENST00000380943
-Accession Number: ENST00000380943
-ID tumour: 2128605
-let_Gene CDS length: 4116
-HGNC ID: NA
-let_Sample_name: S02330
-ID sample: 2261620
-Primary site: lung
-Mutation AA: p.D604Y
-Mutation Description: Substitution - Missense
-Mutation CDS: c.1810G>T
-GRCh: 38
-Mutation genome position: 5:66048688-66048688
-FATHMM prediction: PATHOGENIC
-FATHMM score: 0.99397
-Mutation somatic status: Confirmed somatic variant
-Sample Type: surgery fresh/frozen
-Tumour origin: NS

zahmed@ifh.rutgers.edu

Menu

© Design and developed by Dr. Zeeshan Ahmed

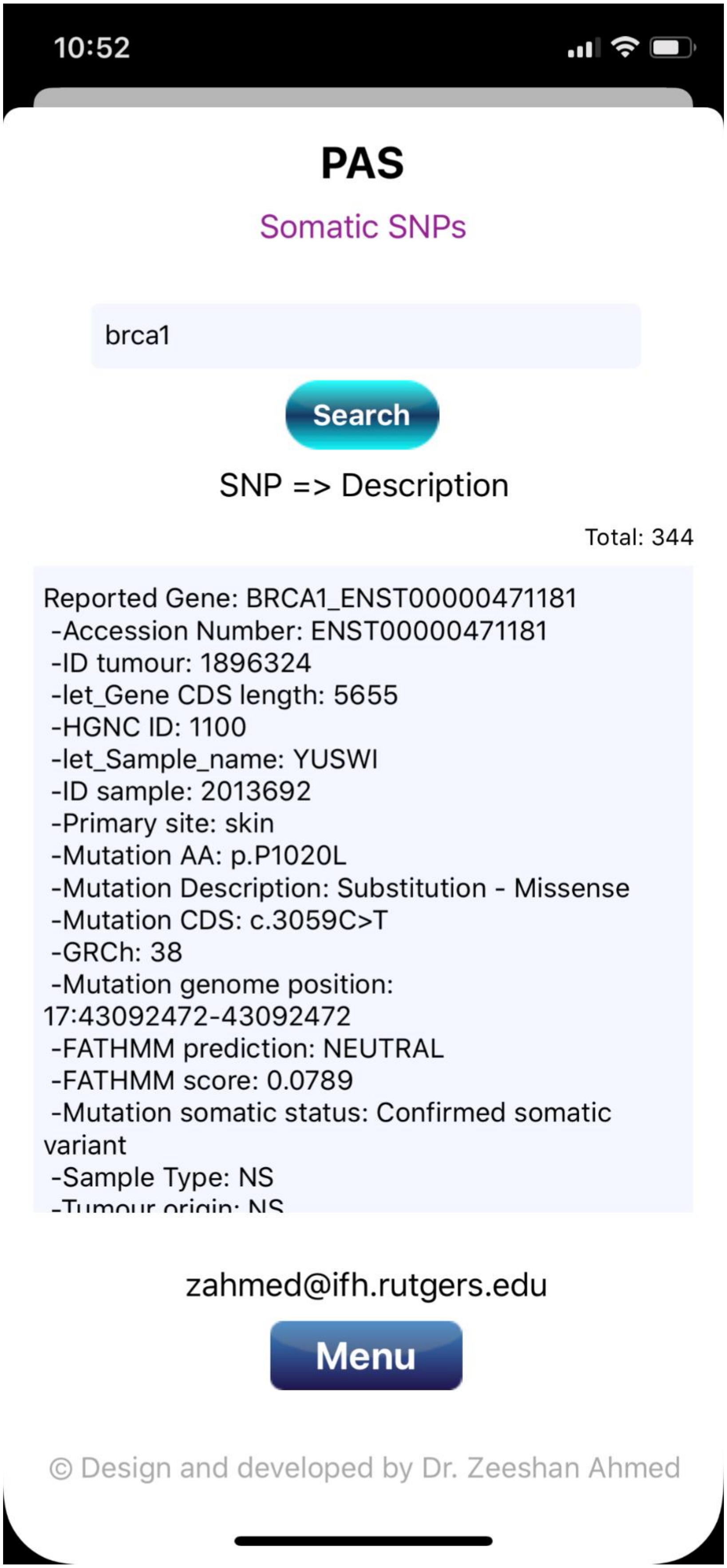
10:52
PAS
Somatic SNPs
brca1
Search
SNP => Description
Total: 344
Reported Gene: BRCA1_ENST00000471181
-Accession Number: ENST00000471181
-ID tumour: 1896324
-let_Gene CDS length: 5655
-HGNC ID: 1100
-let_Sample_name: YUSWI
-ID sample: 2013692
-Primary site: skin
-Mutation AA: p.P1020L
-Mutation Description: Substitution - Missense
-Mutation CDS: c.3059C>T
-GRCh: 38
-Mutation genome position: 17:43092472-43092472
-FATHMM prediction: NEUTRAL
-FATHMM score: 0.0789
-Mutation somatic status: Confirmed somatic variant
-Sample Type: NS
-Tumour origin: NS
zahmed@ifh.rutgers.edu
Menu
© Design and developed by Dr. Zeeshan Ahmed

10:53

PAS

Somatic SNPs

brca2

Search

SNP => Description

Total: 574

Reported Gene: BRCA2
-Accession Number: ENST00000380152
-ID tumour: 2583835
-let_Gene CDS length: 10257
-HGNC ID: 1101
-let_Sample_name: P-0009012-T01-IM5
-ID sample: 2725014
-Primary site: large_intestine
-Mutation AA: p.?
-Mutation Description: Unknown
-Mutation CDS: c.67+1G>T
-GRCh: 38
-Mutation genome position: 13:32316528-32316528
-FATHMM prediction: PATHOGENIC
-FATHMM score: 0.89926
-Mutation somatic status: Confirmed somatic variant
-Sample Type: surgery-fixed
-Tumour origin: primary

zahmed@ifh.rutgers.edu

Menu

© Design and developed by Dr. Zeeshan Ahmed

10:53

PAS

Somatic SNPs

rbm10

Search

SNP => Description

Total: 125

Reported Gene: RBM10
-Accession Number: ENST00000377604
-ID tumour: 2583201
-let_Gene CDS length: 2793
-HGNC ID: 9896
-let_Sample_name: P-0008234-T01-IM5
-ID sample: 2724380
-Primary site: urinary_tract
-Mutation AA: p.S43*
-Mutation Description: Substitution - Nonsense
-Mutation CDS: c.128C>G
-GRCh: 38
-Mutation genome position: 23:47169425-47169425
-FATHMM prediction: PATHOGENIC
-FATHMM score: 0.91231
-Mutation somatic status: Confirmed somatic variant
-Sample Type: surgery-fixed
-Tumour origin: primary

zahmed@ifh.rutgers.edu

Menu

© Design and developed by Dr. Zeeshan Ahmed

10:53

PAS

Somatic SNPs

ptpn13

Search

SNP => Description

Total: 110

Reported Gene: PTPN13
-Accession Number: ENST00000436978
-ID tumour: 1994471
-let_Gene CDS length: 7473
-HGNC ID: 9646
-let_Sample_name: SS6003312
-ID sample: 2120235
-Primary site: oesophagus
-Mutation AA: p.E1090*
-Mutation Description: Substitution - Nonsense
-Mutation CDS: c.3268G>T
-GRCh: 38
-Mutation genome position: 4:86758304-86758304
-FATHMM prediction: PATHOGENIC
-FATHMM score: 0.99341
-Mutation somatic status: Confirmed somatic variant
-Sample Type: NS
-Tumour origin: NS

zahmed@ifh.rutgers.edu

Menu

© Design and developed by Dr. Zeeshan Ahmed

10:53

PAS

Somatic SNPs

ppp6c

Search

SNP => Description

Total: 71

Reported Gene: PPP6C_ENST00000451402
-Accession Number: ENST00000451402
-ID tumour: 2066414
-let_Gene CDS length: 1029
-HGNC ID: 9323
-let_Sample_name: TCGA-BR-8591-01
-ID sample: 2198136
-Primary site: stomach
-Mutation AA: p.T338T
-Mutation Description: Substitution - coding silent
-Mutation CDS: c.1014G>A
-GRCh: 38
-Mutation genome position: 9:125149688-125149688
-FATHMM prediction: NA
-FATHMM score: 0.64415
-Mutation somatic status: Confirmed somatic variant
-Sample Type: NS
-Tumour origin: NS

zahmed@ifh.rutgers.edu

Menu

© Design and developed by Dr. Zeeshan Ahmed

10:45

PAS

Somatic SNPs

esr1

Search

SNP => Description

Total: 216

Reported Gene: ESR1_ENST00000544394
-Primary site: breast
-Primary histology: carcinoma
-Histology subtype: ductal_carcinoma
-Mutation Description: Substitution - Missense
-Mutation genome position: 6:152011697-152011697
-Accession Number: ENST00000544394

Reported Gene: ESR1
-Primary site: large_intestine
-Primary histology: carcinoma
-Histology subtype: adenocarcinoma
-Mutation Description: Substitution - coding silent
-Mutation genome position: 6:151808173-151808173
-Accession Number: ENST00000440973
----

zahmed@ifh.rutgers.edu

Menu

© Design and developed by Dr. Zeeshan Ahmed

10:46

PAS

Somatic SNPs

akt1

Search

SNP => Description

Total: 178

Reported Gene: AKT1
-Primary site: breast
-Primary histology: carcinoma
-Histology subtype: NS
-Mutation Description: Substitution - Missense
-Mutation genome position: 14:104780214-104780214
-Accession Number: ENST00000349310

Reported Gene: AKT1
-Primary site: bone
-Primary histology: Overgrowth_syndrome
-Histology subtype: Proteus_syndrome
-Mutation Description: Substitution - Missense
-Mutation genome position: 14:104780214-104780214
-Accession Number: ENST00000349310
----

zahmed@ifh.rutgers.edu

Menu

© Design and developed by Dr. Zeeshan Ahmed
