# Supplementary material for: Human gene and disease associations for clinical‐genomics and precision medicine research
Source: Clin Transl Med. 2020 May 3;10(1):297–318. doi: 10.1002/ctm2.28 (PMC7240856; doi:10.1002/ctm2.28)
Supplement: Supplementary file 5 — Additional file 5: “Supplementary Figure 6.” [file CTM2-10-297-s005.pdf]

10:20

PAS

International Classification of Diseases (ICD)

diabetes

Search

(ICD) Number => Description

Total: 577

(ICD10) E0800 => Diabetes mellitus due to underlying condition with hyperosmolarity without nonketotic hyperglycemic-hyperosmolar coma (NKHHC)

(ICD10) E0801 => Diabetes mellitus due to underlying condition with hyperosmolarity with coma

(ICD10) E0810 => Diabetes mellitus due to underlying condition with ketoacidosis without coma

(ICD10) E0811 => Diabetes mellitus due to underlying condition with ketoacidosis with coma

(ICD10) E0821 => Diabetes mellitus due to underlying condition with diabetic nephropathy

(ICD10) E0822 => Diabetes mellitus due to

zahmed@ifh.rutgers.edu

Menu

© Design and developed by Dr. Zeeshan Ahmed

10:21

PAS

ICD9

diabetes

Search

(ICD) Number => Description

Total: 69

(ICD9) 24900 => Secondary diabetes mellitus without mention of complication, not stated as uncontrolled, or unspecified

(ICD9) 24901 => Secondary diabetes mellitus without mention of complication, uncontrolled

(ICD9) 24910 => Secondary diabetes mellitus with ketoacidosis, not stated as uncontrolled, or unspecified

(ICD9) 24911 => Secondary diabetes mellitus with ketoacidosis, uncontrolled

(ICD9) 24920 => Secondary diabetes mellitus with hyperosmolarity, not stated as uncontrolled, or unspecified

(ICD9) 24921 => Secondary diabetes mellitus with hyperosmolarity, uncontrolled

zahmed@ifh.rutgers.edu

Menu

© Design and developed by Dr. Zeeshan Ahmed

10:21

PAS

ICD10

diabetes

Search

(ICD) Number => Description

Total: 508

(ICD10) E0800 => Diabetes mellitus due to underlying condition with hyperosmolarity without nonketotic hyperglycemic-hyperosmolar coma (NKHHC)

(ICD10) E0801 => Diabetes mellitus due to underlying condition with hyperosmolarity with coma

(ICD10) E0810 => Diabetes mellitus due to underlying condition with ketoacidosis without coma

(ICD10) E0811 => Diabetes mellitus due to underlying condition with ketoacidosis with coma

(ICD10) E0821 => Diabetes mellitus due to underlying condition with diabetic nephropathy

(ICD10) E0822 => Diabetes mellitus due to

zahmed@ifh.rutgers.edu

Menu

© Design and developed by Dr. Zeeshan Ahmed

10:22

PAS

National Drug Codes (NDC)

diabetes

Search

NDC => Description

Total: 6

NDC: 0372-0043
-TYPE: HUMAN OTC DRUG
-PROPRIETARY NAME: Scot-Tussin Diabetes Cough Formula with DM
-NON PROPRIETARY NAME: dextromethorphan hydrobromide
-DOSAGE FORM NAME: LIQUID
-ROUTE NAME: ORAL
-MARKETING CATEGORY NAME: OTC MONOGRAPH FINAL
-APPLICATION NUMBER: part341
-LABELER NAME: SCOT-TUSSIN Pharmacal Co., Inc.
-SUBSTANCE NAME: DEXTROMETHORPHAN HYDROBROMIDE
-ACTIVE NUMERATOR STRENGTH: 10
-ACTIVE INGRED UNIT: mg/5mL
-PHARM CLASSES: NA
-LISTING RECORD CERTIFIED THROUGH: 20181231

zahmed@ifh.rutgers.edu

Menu

© Design and developed by Dr. Zeeshan Ahmed

10:22

PAS

International Classification of Diseases (ICD)

influenza

Search

(ICD) Number => Description

Total: 44

(ICD10) A413 => Sepsis due to Hemophilus influenzae

(ICD10) A492 => Hemophilus influenzae infection, unspecified site

(ICD10) B963 => Hemophilus influenzae [H. influenzae] as the cause of diseases classified elsewhere

(ICD10) J09X1 => Influenza due to identified novel influenza A virus with pneumonia

(ICD10) J09X2 => Influenza due to identified novel influenza A virus with other respiratory manifestations

(ICD10) J09X3 => Influenza due to identified novel influenza A virus with gastrointestinal manifestations

zahmed@ifh.rutgers.edu

Menu

© Design and developed by Dr. Zeeshan Ahmed

10:22

PAS

ICD9

influenza

Search

(ICD) Number => Description

Total: 16

(ICD9) 03841 => Septicemia due to hemophilus influenzae [H. influenzae]

(ICD9) 0415 => Hemophilus influenzae [H. influenzae] infection in conditions classified elsewhere and of unspecified site

(ICD9) 4802 => Pneumonia due to parainfluenza virus

(ICD9) 4822 => Pneumonia due to Hemophilus influenzae [H. influenzae]

(ICD9) 4870 => Influenza with pneumonia

(ICD9) 4871 => Influenza with other respiratory manifestations

(ICD9) 4878 => Influenza with other manifestations

zahmed@ifh.rutgers.edu

Menu

© Design and developed by Dr. Zeeshan Ahmed

10:23

PAS

ICD10

influenza

Search

(ICD) Number => Description

Total: 28

(ICD10) A413 => Sepsis due to Hemophilus influenzae

(ICD10) A492 => Hemophilus influenzae infection, unspecified site

(ICD10) B963 => Hemophilus influenzae [H. influenzae] as the cause of diseases classified elsewhere

(ICD10) J09X1 => Influenza due to identified novel influenza A virus with pneumonia

(ICD10) J09X2 => Influenza due to identified novel influenza A virus with other respiratory manifestations

(ICD10) J09X3 => Influenza due to identified novel influenza A virus with gastrointestinal manifestations

zahmed@ifh.rutgers.edu

Menu

© Design and developed by Dr. Zeeshan Ahmed

10:23

PAS

National Drug Codes (NDC)

influenza

Search

NDC => Description

Total: 14

NDC: 43742-0235
-TYPE: HUMAN OTC DRUG
-PROPRIETARY NAME: Influenza Grippe Nosode
-NON PROPRIETARY NAME: Influenzinum 2012, Respiratory Syncytial Virus
-DOSAGE FORM NAME: LIQUID
-ROUTE NAME: ORAL
-MARKETING CATEGORY NAME: UNAPPROVED HOMEOPATHIC
-APPLICATION NUMBER: NA
-LABELER NAME: Deseret Biologicals, Inc.
-SUBSTANCE NAME: INFLUENZA A VIRUS A/CALIFORNIA/7/2009 X-179A (H1N1) ANTIGEN (FORMALDEHYDE INACTIVATED); INFLUENZA A VIRUS A/PERTH/16/2009 (H3N2) LIVE (ATTENUATED) ANTIGEN; INFLUENZA B VIRUS B/BRISBANE/60/2008 ANTIGEN (FORMALDEHYDE INACTIVATED); RESPIRATORY SYNCYTIAL VIRUS IMMUNE GLOBULIN INTRAVENOUS (HUMAN)
-ACTIVE NUMERATOR STRENGTH: 9; 9; 9; 8

zahmed@ifh.rutgers.edu

Menu

© Design and developed by Dr. Zeeshan Ahmed

10:23

PAS

International Classification of Diseases (ICD)

fever

Search

(ICD) Number => Description

Total: 110

(ICD10) A0100 => Typhoid fever, unspecified

(ICD10) A0102 => Typhoid fever with heart involvement

(ICD10) A0109 => Typhoid fever with other complications

(ICD10) A011 => Paratyphoid fever A

(ICD10) A012 => Paratyphoid fever B

(ICD10) A013 => Paratyphoid fever C

(ICD10) A014 => Paratyphoid fever, unspecified

(ICD10) A259 => Rat-bite fever, unspecified

(ICD10) A380 => Scarlet fever with otitis media

zahmed@ifh.rutgers.edu

Menu

© Design and developed by Dr. Zeeshan Ahmed

10:23

PAS

ICD9

fever

Search

(ICD) Number => Description

Total: 48

(ICD9) 0020 => Typhoid fever

(ICD9) 0021 => Paratyphoid fever A

(ICD9) 0022 => Paratyphoid fever B

(ICD9) 0023 => Paratyphoid fever C

(ICD9) 0029 => Paratyphoid fever, unspecified

(ICD9) 0260 => Spirillary fever

(ICD9) 0261 => Streptobacillary fever

(ICD9) 0269 => Unspecified rat-bite fever

(ICD9) 0341 => Scarlet fever

(ICD9) 0600 => Sylvatic yellow fever

zahmed@ifh.rutgers.edu

Menu

© Design and developed by Dr. Zeeshan Ahmed

10:24

PAS

ICD10

fever

Search

(ICD) Number => Description

Total: 62

(ICD10) A0100 => Typhoid fever, unspecified

(ICD10) A0102 => Typhoid fever with heart involvement

(ICD10) A0109 => Typhoid fever with other complications

(ICD10) A011 => Paratyphoid fever A

(ICD10) A012 => Paratyphoid fever B

(ICD10) A013 => Paratyphoid fever C

(ICD10) A014 => Paratyphoid fever, unspecified

(ICD10) A259 => Rat-bite fever, unspecified

(ICD10) A380 => Scarlet fever with otitis media

zahmed@ifh.rutgers.edu

Menu

© Design and developed by Dr. Zeeshan Ahmed

10:24

PAS

National Drug Codes (NDC)

fever

Search

NDC => Description

Total: 284

NDC: 0113-0020
-TYPE: HUMAN OTC DRUG
-PROPRIETARY NAME: good sense childrens pain and fever
-NON PROPRIETARY NAME: Acetaminophen
-DOSAGE FORM NAME: SUSPENSION
-ROUTE NAME: ORAL
-MARKETING CATEGORY NAME: OTC MONOGRAPH NOT FINAL
-APPLICATION NUMBER: part343
-LABELER NAME: L. Perrigo Company
-SUBSTANCE NAME: ACETAMINOPHEN
-ACTIVE NUMERATOR STRENGTH: 160
-ACTIVE INGRED UNIT: mg/5mL
-PHARM CLASSES: NA
-LISTING RECORD CERTIFIED THROUGH: 20191231
-PRODUCT ID: 0113-0020_fe2d2ffa-f511-4451-8911-4e1102c25ccc
----

zahmed@ifh.rutgers.edu

Menu

© Design and developed by Dr. Zeeshan Ahmed

10:25

PAS

International Classification of Diseases (ICD)

sterile

Search

(ICD) Number => Description

Total: 18

(ICD10) Y620 => Failure of sterile precautions during surgical operation

(ICD10) Y621 => Failure of sterile precautions during infusion or transfusion

(ICD10) Y622 => Failure of sterile precautions during kidney dialysis and other perfusion

(ICD10) Y623 => Failure of sterile precautions during injection or immunization

(ICD10) Y624 => Failure of sterile precautions during endoscopic examination

(ICD10) Y625 => Failure of sterile precautions during heart catheterization

(ICD10) Y626 => Failure of sterile precautions during aspiration, puncture and other [illegible]

zahmed@ifh.rutgers.edu

Menu

© Design and developed by Dr. Zeeshan Ahmed

10:26

PAS

ICD9

sterile

Search

(ICD) Number => Description

Total: 9

(ICD9) E8720 => Failure of sterile precautions during surgical operation

(ICD9) E8721 => Failure of sterile precautions during infusion or transfusion

(ICD9) E8722 => Failure of sterile precautions during kidney dialysis and other perfusion

(ICD9) E8723 => Failure of sterile precautions during injection or vaccination

(ICD9) E8724 => Failure of sterile precautions during endoscopic examination

(ICD9) E8725 => Failure of sterile precautions during aspiration of fluid or tissue, puncture, and catheterization

(ICD9) E8726 => Failure of sterile precautions

zahmed@ifh.rutgers.edu

Menu

© Design and developed by Dr. Zeeshan Ahmed

10:26

PAS

ICD10

sterile

Search

(ICD) Number => Description

Total: 9

(ICD10) Y620 => Failure of sterile precautions during surgical operation

(ICD10) Y621 => Failure of sterile precautions during infusion or transfusion

(ICD10) Y622 => Failure of sterile precautions during kidney dialysis and other perfusion

(ICD10) Y623 => Failure of sterile precautions during injection or immunization

(ICD10) Y624 => Failure of sterile precautions during endoscopic examination

(ICD10) Y625 => Failure of sterile precautions during heart catheterization

(ICD10) Y626 => Failure of sterile precautions during aspiration, puncture and other

zahmed@ifh.rutgers.edu

Menu

© Design and developed by Dr. Zeeshan Ahmed

10:26

PAS

National Drug Codes (NDC)

sterile

Search

NDC => Description

Total: 138

NDC: 0002-0800
-TYPE: HUMAN OTC DRUG
-PROPRIETARY NAME: Sterile Diluent
-NON PROPRIETARY NAME: diluent
-DOSAGE FORM NAME: INJECTION, SOLUTION
-ROUTE NAME: SUBCUTANEOUS
-MARKETING CATEGORY NAME: NDA
-APPLICATION NUMBER: NDA018781
-LABELER NAME: Eli Lilly and Company
-SUBSTANCE NAME: WATER
-ACTIVE NUMERATOR STRENGTH: 1
-ACTIVE INGRED UNIT: mL/mL
-PHARM CLASSES: NA
-LISTING RECORD CERTIFIED THROUGH: 20191231
-PRODUCT ID: 0002-0800_4bb5d1cb-0fa7-48c7-9f6d-8d45f9b91649
----

zahmed@ifh.rutgers.edu

Menu

© Design and developed by Dr. Zeeshan Ahmed
